# Deep learning for cardiovascular disease: a comprehensive review of detection and risk forecasting

**DOI:** 10.3389/frai.2026.1840804

**Published:** 2026-06-26

**Authors:** N. Ganeshan, G. Magesh

**Affiliations:** School of Computer Science Engineering and Information Systems, Vellore Institute of Technology, Vellore, India

**Keywords:** BiGRU-Attention, cardiovascular disease, deep learning, explainability, federated learning, IoT healthcare, multimodal data fusion, risk prediction

## Abstract

The biggest health threat to the global population is cardiovascular disease (CVD), which afflicts almost one-third of the global population and causes considerable monetary and social losses. Risk analysis should be performed in a timely and appropriate manner to enhance clinical practice and preventive interventions. The emergence of advanced data modalities, such as wearable sensors, medical imaging, electronic health records (EHRs), and genomic platforms, has led to a paradigm shift in the holistic assessment of CVD risk through multimodal data integration. This systematic review is a methodological analysis of recent multimodal input deep-learning algorithms that enhance the early detection of CVD and risk-specific evaluation, following PRISMA 2020 guidelines across 69 studies published 20,122,025 based on 2,847 initial database records. We characterized the wide range of available data streams: longitudinal physiological measurements (ECG, HRV, and BP), echocardiogram data, cardiac MRI and CT, lab/demographic data, behavioral/environmental data, and genomic/proteomic data. Mid-level, early, late, and attention-based fusion methods are described in the context of deep neural networks, such as CNNs, RNNs, BiGRU with attention, and hybrid CNN-LSTM networks. Comparative studies showed dramatic improvements in predictive accuracy (often over 98%), strength to missing or noisy modalities, and access to real-time, individualized recommendations. The best-performing DEEP-CARDIO BiGRU-Attention model had 99.9 percent accuracy on Framingham and Statlog benchmarks. A systematic review of 28 studies by Grad-CAM and SHAP confirmed the dominance of each in imaging and structured-data tasks, respectively (Rahman et al., 2024). The federated explainable FL-LSTM model achieved 99% AUC across three ECG databanks with complete privacy protection. We end with a systematic reproducibility, federated learning, equitable AI, and regulatory translation roadmap.

## 1 Introduction

**Figure 1:**
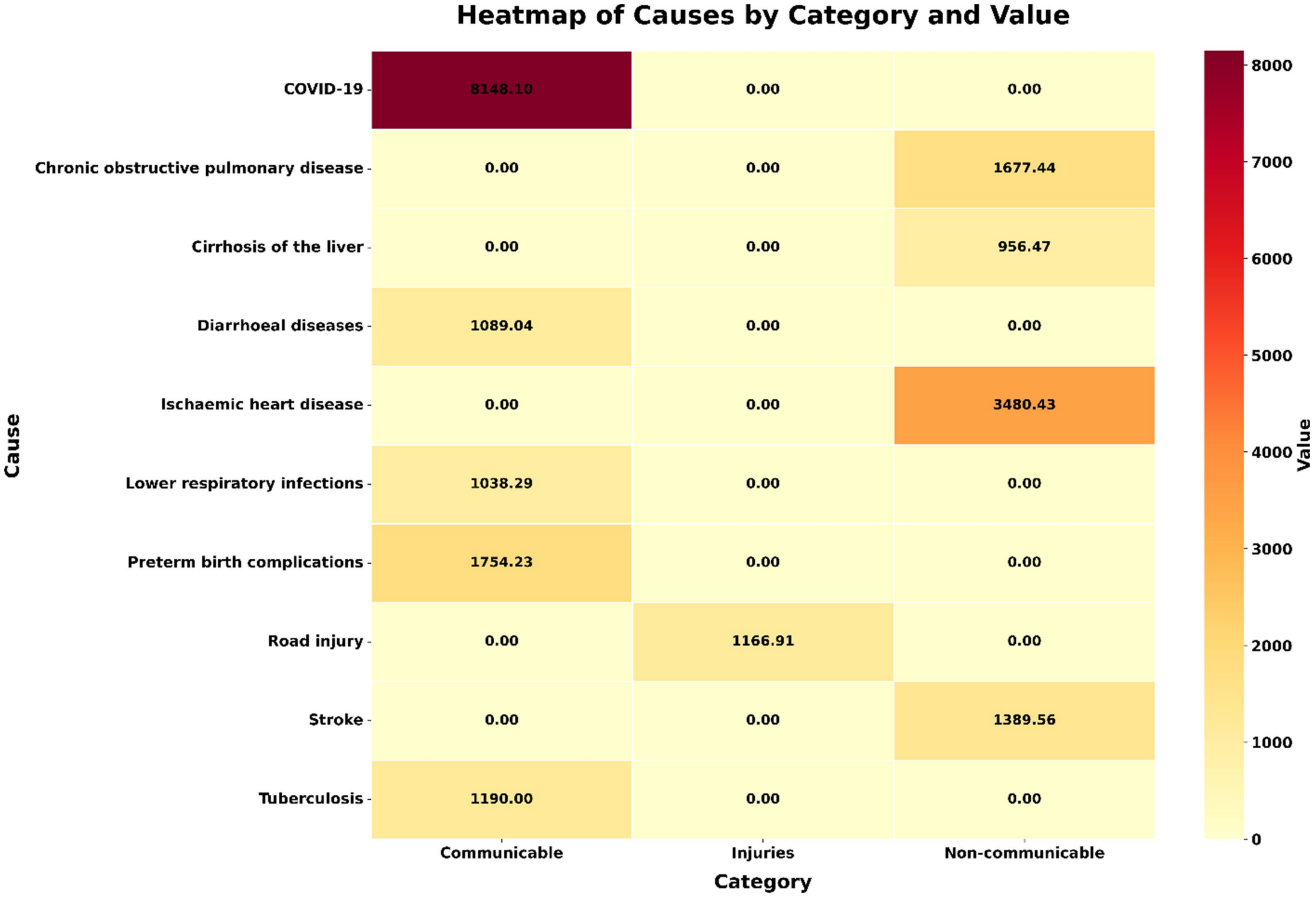
WHO-Reported Global Cardiovascular Disease Mortality Heatmap by Country. [Adapted. Citation: Global Health Estimates 2021: Deaths by Cause, Age, Sex, by Country and by Region, 2000–2021. Geneva, World Health Organization; 2024. License: CC BY-NC-SA 3.0 IGO. Source: https://www.who.int/data/gho/data/themes/mortality-and-global-health-estimates/ghe-leading-causes-of-death].

Cardiovascular diseases (CVDs) are a group of multiple disorders of the heart and blood vessels, such as coronary artery disease, stroke, heart failure, arrhythmias, and peripheral artery disease, which is the leading cause of death in the world, with an estimated 17.9 million deaths annually, approximating 32 percent of all deaths globally (Holt et al., 2023; Hathaway et al., 2021; Barbieri et al., 2020). In high-income countries, the burden of CVD has decreased with improved healthcare, whereas in low-to-middle income nations, there is a growing risk burden due to metabolic syndrome, urbanization, sedentary lifestyles, and pollution (Wang et al., 2021; Ordikhani et al., 2022) (Figure 1; Table 1).

**Table 1:** Regional CVD mortality and key risk factors.

Region	CVD mortality (per 100,000)	Most common subtype	Notable risk factor prevalence
North America	180	Ischaemic Heart Disease	Hypertension (35%), Obesity (41%)
Western Europe	160	Ischaemic Heart Disease	High cholesterol (33%)
East Asia	205	Stroke	Smoking (28%), Salt intake (43%)
South Asia	260	Stroke	Diabetes (16%), Hypertension (41%)
Sub-Saharan Africa	250	Hypertensive Heart Disease	Physical inactivity (42%), HIV comorbidity (12%)

**Figure 2:**
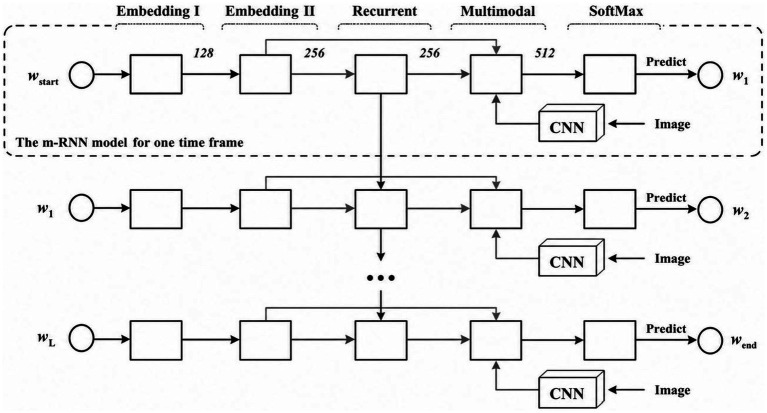
Framework for multimodal cardiovascular disease risk stratification (Gao et al., 2020).

CVDs do not only have a human cost in terms of premature mortality—30 percent before the age of 70—but also have a significant economic cost in terms of direct medical expenses, lost productivity, and social dependency. The socio-demographic change, rising levels of obesity, sedentary lifestyles, poor diets, pollution, and tobacco use have further increased the number of at-risk populations in all age groups and on all continents, making CVD an urgent global public health concern (Bhagawati et al., 2023; Addissouky et al., 2024). The traditional risk models, namely the Framingham Risk Score and the ASCVD calculator, are based on the assumption of a linear relationship and lack temporal dynamics, limiting generalizability, especially in women, ethnic minorities, and individuals with multimorbidity (Yun et al., 2022; Wang et al., 2021; Ordikhani et al., 2022). Wearable sensors, EHR platforms, omics technologies, and IoT infrastructure are proliferating, revolutionizing CVD prediction by generating multimodal data streams that were previously unavailable to deep neural architectures (Sornalakshmi et al., 2024; Ahmad et al., 2021). The multimodal data landscape includes: (1) Clinical data—laboratory values, diagnoses, comorbidities, medications, and family history; (2) Physiological indicators—longitudinal ECG, HRV, blood pressure, and data from wearable monitors; (3) Medical imaging—echocardiograms, cardiac MRI, CT angiography, and vascular data from mammograms; (4) Genomics/omics—polygenic risk scores, transcriptomics, and methylation signatures; (5) Behavioral/environmental—lifestyle, activity, diet, and exposures sampled using smartphones and apps (Figure 2).

A case in point is systems like DEEP-CARDIO, which streamline the use of deep neural architectures for real-time ECG, blood pressure, pulse, and glucose, classifying conditions and providing interventions in real time. Hybrid neural systems combining CNN and LSTM have achieved predictive accuracies over 98%, compared to traditional systems. Decision trees and SVMs are best applied to structured tabular data, whereas ANNs are used to process mixed clinical data, CNNs to medical images, and RNNs (including LSTM and GRU) on the sequential physiological data (Gupta and Singh, 2023; Zhang et al., 2021; Krishnan et al., 2021; Reshan et al., 2023; Almulihi et al., 2022; Vincent et al., 2022; Yashudas et al., 2024).

### 1.1 Literature search strategy and PRISMA flow

**Figure 3:**
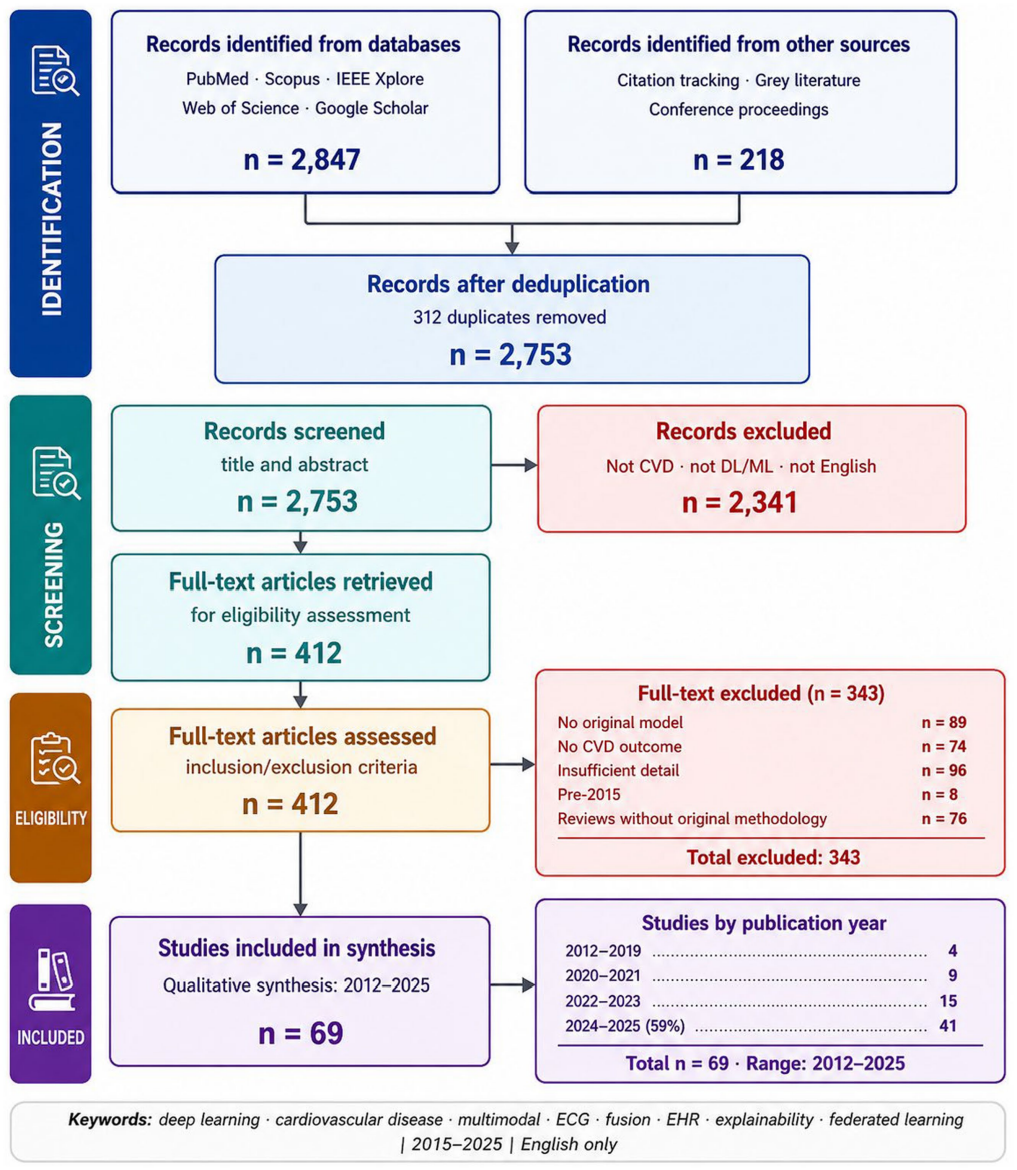
PRISMA 2020 flow diagram—multimodal deep learning for CVD systematic review. Literature identification (*n* = 3,065), screening (*n* = 2,753), eligibility assessment (*n* = 412), and final inclusion (*n* = 69 studies, 2012–2025) (Singh et al., 2024; Rahman et al., 2024).

This review is based on the PRISMA 2020 guidelines. Searches in PubMed, Scopus, IEEE Xplore, Web of Science, and Google Scholar (2015–2025) used Boolean combinations of: deep learning, CVD, multimodal data, ECG, medical imaging, risk prediction, fusion, EHR, wearable sensors, and explainability (Stahlschmidt et al., 2022; Gao et al., 2020). Inclusion criteria: (1) peer-reviewed articles with original DL/ML CVD models; (2) at least one of the following clinical/physiological/imaging/genomic modality; (3) quantitative measures of performance reported. Exclusion criteria: (1) No original model; (2) non-CVD outcomes; (3) lack of methodological detail; (4) non-English; (5) pre-2015 publications; (6) duplicate datasets without new methodology. The first search has yielded 2,847 records in the database and 218 additional sources. After eliminating 312 duplicates, 2,753 records were screened, with 2,341 being eliminated at the title/abstract stage. Out of 412 full-text articles evaluated, 343 were filtered out. Final synthesis: 69 studies, with 59% (*n* = 41) published 2024–2025 (Singh et al., 2024; Rahman et al., 2024) (Figure 3).

A contemporaneous systematic review of 65 ML studies (Banerjee et al., 2025) confirms that despite high benchmark accuracy, a significant translational gap persists—driven by dataset overreliance, lack of external validation, and black-box opacity—underscoring the necessity of the XAI and multimodal integration focus of the present review.

## 2 Multimodal data sources in cardiovascular risk stratification

**Figure 4:**
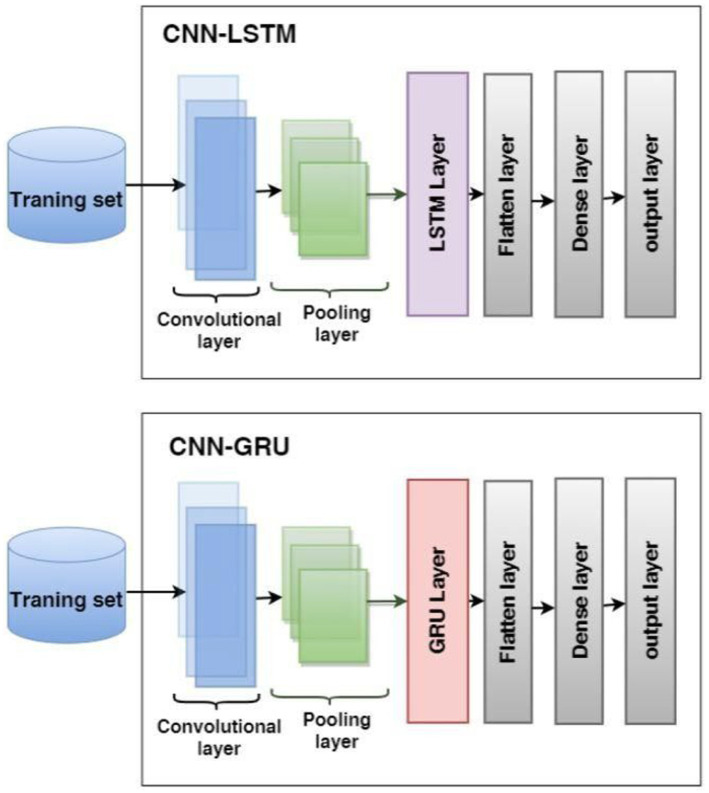
Historical shift in CVD diagnosis: from traditional risk scores to deep learning approaches (Zhang et al., 2023).

The paradigm shift in predicting CVD is based on integrating five complementary data streams that encode different types of physiological information that cannot be captured adequately by any single data stream (Stahlschmidt et al., 2022; Gao et al., 2020; Zhang et al., 2023; Li et al., 2024). EHR data provide consistent, stable demographic bases; wearable signals capture real-time temporal changes; medical imaging provides spatial morphological details; genomics marks lifetime risk factors; and behavioral/environmental data capture lifetime and exposure determinants (Vincent et al., 2022; Yashudas et al., 2024; Holt et al., 2023). At the center of this paradigm is the compilation of very large, high-resolution databases such as UCI Heart, Framingham, MIMIC-III, PhysioNet MIT-BIH, and Cardiology DICOM, which capture various aspects of cardiovascular risk, including demographic and laboratory data, continuous physiological recordings, and advanced imaging (Figure 4; Tables 2, 3).

**Table 2:** Comprehensive cardiovascular dataset profiles—demographics, imaging specifications, annotation standards, and harmonization.

Dataset	Modality	Size and class distribution	Patient demographics	Imaging/signal specs	Annotation standard	Multi-center and harmonization	Access
UCI Heart Disease	Clinical + Labs	303 records; 54.4% disease positive, 45.6% negative	Age 29–77 yrs. (mean 54.4); 68% male, 32% female; mixed US population	14 clinical/lab features; no imaging	Cardiologist-confirmed diagnosis labels	Single-center (Cleveland Clinic); no harmonization applied	archive.ics.uci.edu
Framingham Heart Study	EHR + Longitudinal	4,238 records; ~15% CVD event positive	Age 32–70 yrs.; ~52% female; predominantly White US cohort	Longitudinal clinical variable: SBP, DBP, cholesterol, glucose, BMI, smoking	Physician-adjudicated 10-year CVD event outcomes	Single community cohort (Framingham, MA); standardized exam protocols across waves	kaggle.com/framingham
MIMIC-III	EHR + Time-series	60,000 + ICU admissions; multiclass (cardiac, respiratory, sepsis)	Age 18–90 + yrs.; ~56% male; racially diverse US ICU population	Timestamped vitals (HR, BP, SpO2), labs, ICD-9 codes, waveforms at 125 Hz	ICD-9 coded diagnoses; physician notes	Single institution (Beth Israel Deaconess, Boston); de-identified per HIPAA; no cross-site harmonization	physionet.org
PhysioNet MIT-BIH	ECG	48 half-hour recordings; 17 arrhythmia classes	Age 23–89 yrs.; 22 male, 25 female; mixed ambulatory patients	2-channel ambulatory ECG at 360 Hz; MLII + V5 leads	Beat-level annotation by 2 independent cardiologists; adjudicated consensus labels	Single center; signal standardized to 360 Hz; baseline wander removed	physionet.org
Statlog Heart	Clinical variables	270 records; 55.6% disease, 44.4% healthy	Age and sex distribution similar to UCI; European cohort	13 clinical variables (subset of UCI features)	Binary classification labels; source cardiologist-verified	Multi-source compilation (UCI, Hungarian, Swiss); no explicit harmonization protocol documented	UCI/Statlog
UK Biobank	MRI + CT + Genomics + EHR	30,000 + cardiac MRIs; 500,000 participant cohort	Age 40–69 yrs. at recruitment; ~54% female; predominantly White British	Cardiac MRI: 1.5 T scanner; short-axis cine, long-axis views; standardized DICOM format	Automated + expert-reviewed segmentation; phenotype definitions via ICD-10	Multi-site UK (>20 assessment centers); centralized MRI acquisition protocol; batch correction applied	Application required
EchoNet dynamic	Echocardiography video	10,030 apical 4-chamber echo videos; EF labels	Stanford Hospital patients; diverse age/sex; no explicit race breakdown published	Grayscale AVI video; 112 × 112 pixels; ~34 fps; variable clip length	EF measured by 3 independent cardiologists; mean EF used as ground truth	Single center (Stanford); scanner-normalized preprocessing pipeline provided	echonet.github.io
Cardiology DICOM	Imaging (ECHO, MRI)	Variable; dataset-dependent	Not standardized; varies by contributing institution	DICOM-format cardiac imaging across modalities	Varies; no unified annotation standard	Multi-source; no formal harmonization protocol	kaggle.com

Dataset	Modality	Size	Access	References	Key Features
UCI Heart Disease	Clinical + Labs	303 records	archive.ics.uci.edu	Zhang et al. (2021); Reshan et al. (2023); Almulihi et al. (2022)	14 + features
Framingham Study	EHR + Demographic + Longitudinal	4,238 records	www.kaggle.com/framingham	Holt et al. (2023)	Age, SBP, glucose, HDL, smoking
MIMIC-III	EHR + Time-series Physiology	60,000 + ICU	physionet.org	Hathaway et al. (2021); Barbieri et al. (2020)	ICD codes, labs, treatment
PhysioNet MIT-BIH	ECG (Wearable)	48 recordings	physionet.org	Yashudas et al. (2024); Sumalatha et al. (2024)	Arrhythmia annotations
Statlog Heart	Clinical variables	270–303 records	UCI/Statlog	Yashudas et al. (2024)	Formatted for ML benchmarks
Cardiology DICOM	Imaging (ECHO, MRI)	Varies	kaggle.com/imaging-cardiac	Wong and Tse (2021)	Cardiac imaging modalities
UK Biobank	MRI + CT + Genomics + EHR	30,000 + cardiac MRIs	Application required	Zhang et al. (2023)	Multi-modal; largest open CVD dataset
EchoNet dynamic	Echocardiography video	10,000 + labelled	Fully open	Zhang et al. (2023)	Gold standard for echo-based DL

**Table 3:** Structured cardiovascular data sources (with key features).

Dataset	Modality	Size/samples	Key features	Access	References
UCI Heart Disease	Demographic/labs	303	14 + features	UCI repository	Zhang et al. (2021); Reshan et al. (2023); Almulihi et al. (2022)
Framingham study	Dem, labs, longitudinal	4,238+	Age, gender, SBP, glucose, HDL, smoking	Kaggle	Holt et al. (2023)
MIMIC-III	EHR, labs, codes	60,000+	Time-stamped EHR; ICD, labs, treatment	PhysioNet	Hathaway et al. (2021); Barbieri et al. (2020)
Statlog heart	Clinical variables	270–303	Similar to UCI, formatted for ML	UCI/Statlog	Yashudas et al. (2024)

### 2.1 Clinical and structured data

A basic backbone of CVD risk modeling is provided by EHR and structured data (diagnoses, lab values, vital signs, medication use, procedural history, and family history) (Reshan et al., 2023; Bhagawati et al., 2023). The field is supported by reliable publicly available datasets such as UCI Heart Disease (303 records, 14 + features), Framingham Study (4,238 + records), and MIMIC-III (60,000 + ICU admissions) (Table 4).

**Table 4:** Clinical parameters and medical relevance in CVD prediction.

Parameter	Measurement/encoding	Medical relevance	Source example	References
Age	Years (integer)	Baseline CVD risk; interacts with most risk factors	UCI Heart, Framingham	Zhang et al. (2021); Holt et al. (2023)
Sex/gender	Male/female (binary)	Men with higher risk earlier; women with post-menopause	All datasets	Zhang et al. (2021); Holt et al. (2023); Wang et al. (2021)
BP (SBP/DBP)	mmHg (continuous)	Hypertension is a primary modifiable CVD risk factor	UCI, Framingham	Zhang et al. (2021); Holt et al. (2023)
Diabetes mellitus	Yes/No	Multiplies CVD risk 2-4x	All datasets	Selvarathi and Varadhaganapathy (2023); Wang et al. (2021)
Cholesterol (LDL, HDL, total)	mg/dL or mmol/L	Key predictor of atherosclerosis progression	UCI, Framingham	Zhang et al. (2021); Holt et al. (2023)
family history	Binary	Captures polygenic and monogenic inherited risk	UCI Heart, Framingham	Zhang et al. (2021); Holt et al. (2023)

### 2.2 Physiological signals and wearable data

Real-time temporal variations, which are not captured by clinical data alone, are provided by physiological signals, namely ECG (at-rest ambulatory), HRV, continuous blood pressure, glucose monitoring, and SpO2 (Vincent et al., 2022; Holt et al., 2023; Krittanawong et al., 2019; Haq et al., 2020). State-of-the-art neural networks, such as RNN, LSTM, GRU, BiLSTM, and BiGRU with attention, have been confirmed on large datasets and have demonstrated exceptional performance in detecting subtle arrhythmias and impending CVD events (Table 5).

**Table 5:** Physiological signals and deep learning approaches for CVD detection.

Signal	Key features	Physiological significance	Dataset(s)	Advantage	Limitation	References
ECG	PQRST intervals, arrhythmia type	Cardiac electrophysiology, arrhythmia risk	MIT-BIH, DEEP-CARDIO	High temporal resolution	Noise/electrode sensitivity	Yashudas et al. (2024); Sumalatha et al. (2024)
HRV	RMSSD, LF/HF, SDNN	Autonomic tone, stress markers	MIMIC-III, PhysioNet	Non-invasive autonomic marker	External factors reduce specificity	Yashudas et al. (2024); Sumalatha et al. (2024)
BP (beat-to-beat)	SBP/DBP, variability index	Hypertension and vascular stiffness	DEEP-CARDIO, MIMIC-III	Key CVD indicator via wearables	Calibration required	Sornalakshmi et al. (2024); Yashudas et al. (2024)
SpO2	% saturation, variability	Oxygenation, sleep apnea risk	MIMIC-III, DEEP-CARDIO	Simple continuous monitoring	Low specificity; altitude sensitive	Sornalakshmi et al. (2024); Yashudas et al. (2024)
Glucose	Level, postprandial excursions	Diabetes-CVD comorbidity	IoT, DEEP-CARDIO	Strong metabolic CVD signal	Requires frequent monitoring	Sornalakshmi et al. (2024); Yashudas et al. (2024); Selvarathi and Varadhaganapathy (2023)

The BiGRU-Attention method of the DEEP-CARDIO system was a 99.9% accurate multiclass CVD detector that used simultaneous biosensor data, a significant improvement over classical machine learning or single sensor methods (Krittanawong et al., 2019). The ESMO-optimized EAWO-DNN, by incorporating ESMO optimization, increased network lifetime and diagnostic sensitivity by guaranteeing efficient power clustering in IoT systems (An et al., 2019) (Table 6).

**Table 6:** Overview of cardiovascular imaging techniques, AI models, and data resources.

Imaging type/model	Parameters/use case	Medical utility/strength	Dataset/Source	Contribution	Limitation	References
Echocardiogram	Chamber volumes, EF, wall motion, strain	Heart failure, hypertrophy, structural analysis	EchoNet Dynamic	Fine-grained, interpretable risk markers	Inter-operator variability	Wehbe et al. (2023); Amal et al. (2024)
CCTA (CT Angiography)	Coronary calcium (CAC), plaque, stenosis	Atherosclerosis quantification	Kaggle CCTA, PACS	Quantifies atherosclerosis and valve disease	High imaging cost	Barbieri et al. (2020); Cocianu et al. (2023)
Cardiac MRI	Fibrosis, volumes, perfusion, functional indices	Myocarditi, HF viability assessment	UK Biobank	Radiomics extracts hidden predictive markers	High storage requirements	Selvarathi and Varadhaganapathy (2023); Sekar et al. (2012)
Mammogram (BAC)	Breast arterial calcification, calcium mass	Predicts coronary artery disease	BAC datasets, Wang et al.	AI BAC detection matches expert-level performance	Limited generalizability	Selvarathi and Varadhaganapathy (2023); Yun et al. (2022)
CNN (2D/3D)	Heart/artery segmentation, image classification	Learns spatial and anatomical structures efficiently	EchoNet, CMR datasets	Deep spatial learning	Requires many labeled images	Sumalatha et al. (2024); Selvarathi and Varadhaganapathy (2023); Yun et al. (2022)
U-Net/DenseUNet	Precise tissue and lesion segmentation	High-resolution ROI handling	EchoNet, CCTA	Robust segmentation in noisy imaging	Needs GPU resources	Zhang et al. (2023); Selvarathi and Varadhaganapathy (2023)
ResNet/inception	Plaque and calcification detection	Transfer learning; excellent classification	Kaggle, CCTA	Deeper networks generalize better	Large model size	Zhang et al. (2023); Yun et al. (2022)
Autoencoder + CNN	Dimensionality reduction and BAC classification	Lowers feature complexity; enhances performance	Mammogram BAC datasets	Improves AUC with fewer features	Risk of overfitting	Yun et al. (2022)
CNN (2D/3D)	Efficient heart/artery segmentation and image classification of spatial and anatomical structures	Learns spatial and anatomical structures efficiently	EchoNet, CMR datasets	Deep spatial learning	Requires many labeled images	Sumalatha et al. (2024); Selvarathi and Varadhaganapathy (2023); Yun et al. (2022); Wani et al. (2024)
Mammogram (BAC)	Breast arterial calcification, calcium mass	Predicts coronary artery disease	BAC datasets, Wang et al.	AI BAC detection matches expert-level performance	Limited generalizability	Selvarathi and Varadhaganapathy (2023); Yun et al. (2022); Alzahrani et al. (2025)

### 2.3 Medical imaging and radiomics

The imaging-based cardiovascular biomarkers, echocardiography, cardiac magnetic resonance (CMR), coronary computed tomography angiography (CCTA), and mammograms of breast arterial calcification (BAC) allow the knowledge of the spatial, structural, and functional parameters of the cardiovascular system (Gupta and Singh, 2023; Holt et al., 2023; Zhang et al., 2023; Yun et al., 2022). These imaging modalities can give direct, quantifiable measurements of myocardial phenotypes, including chamber size, ejection fraction, vascular wall thickness, plaque burden, and calcification patterns.

Integration of genomics, proteomics, behavioral, and environmental data enables the identification of rare and complex causes, lifetime risk markers, inflammation markers, and social determinants of CVD (Krishnan et al., 2021; Wong and Tse, 2021; Yun et al., 2022). Major repositories such as the UK Biobank and dbGaP support this line of research.

### 2.4 Genomics, proteomics, and multi-omics

**Figure 5:**
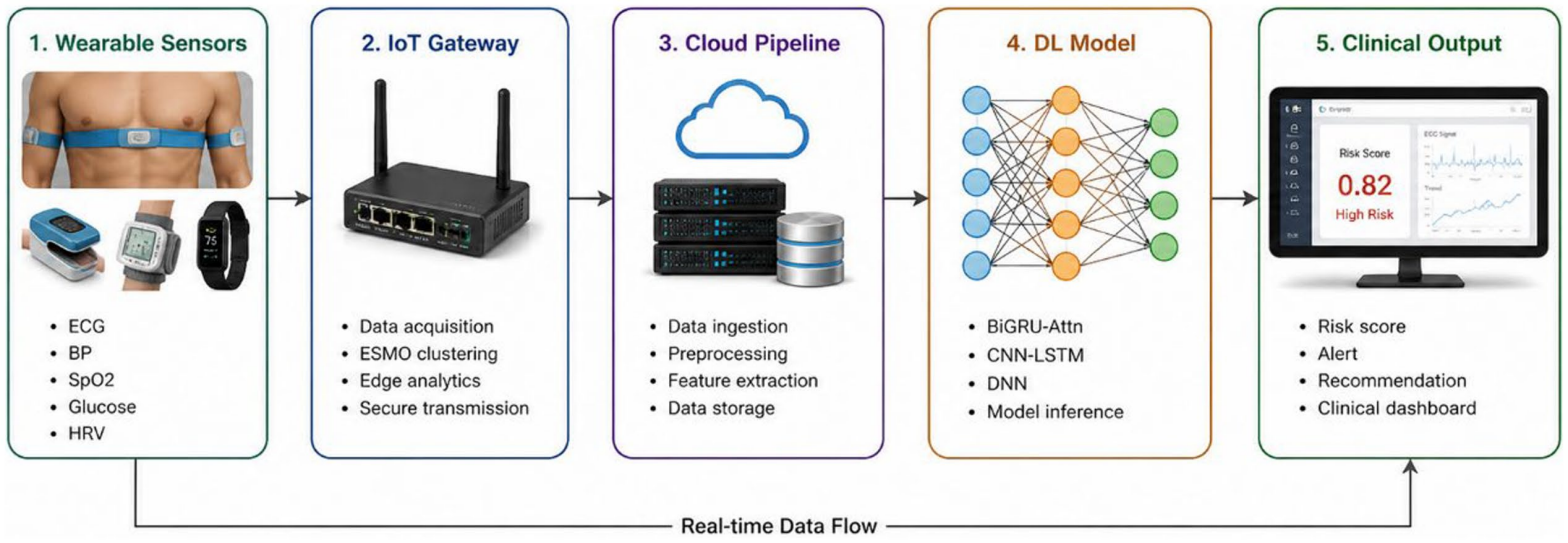
IoT-cloud-edge deep learning pipeline for real-time multimodal CVD risk detection—end-to-end architecture from wearable sensor acquisition through cloud-based DL fusion to clinician-facing recommendation (Sornalakshmi et al., 2024; Yashudas et al., 2024; Stahlschmidt et al., 2022; Mulani et al., 2025).

Integration of genomics, proteomics, behavioral, and environmental data enables the identification of rare and complex causes, lifetime risk markers, inflammatory markers, and social determinants of CVD (Krishnan et al., 2021; Wong and Tse, 2021; Yun et al., 2022). Major repositories such as the UK Biobank and dbGaP support this direction of research (Figure 5) (Tables 7, 8).

**Table 7:** Multi-omics biomarkers in CVD risk assessment.

Type	Parameter/definition	Predictive role	Key dataset(s)
SNP/Genetic loci	Polygenic/monogenic variant alleles	Stable, lifetime CVD risk	UK Biobank, dbGaP (Hathaway et al., 2021)
DNA methylation	CpG site status, methylation clocks	Age-independent, epigenetic risk	dbGaP, TCGA (Yun et al., 2022)
RNAseq/Transcriptomics	mRNA, lncRNA, miRNA, etc.	Inflammatory at-risk phenotype	TCGA (Yun et al., 2022)
Proteomics	Cardiac/vascular protein levels	Biomarker of ongoing disease	Frontiers Cardiovascular Medicine (Mulani et al., 2025)

**Table 8:** Overview of major multimodal data types, features, and datasets.

Data type	Typical features	Example dataset	Access	Reference
Demographics/EHR	Age, sex, BP, cholesterol, comorbidities, ICD codes	UCI Heart, Framingham, MIMIC-III	Public/Kaggle	Zhang et al. (2021); Reshan et al. (2023); Almulihi et al. (2022)
Wearable signals	ECG, HRV (RMSSD, LF/HF), BP, SpO2, glucose	MIT-BIH, PhysioNet, DEEP-CARDIO	PhysioNet	Vincent et al. (2022); Yashudas et al. (2024); Holt et al. (2023)
Medical imaging	ECHO (EF, strain), cardiac MRI, CCTA (CAC, plaque)	EchoNet Dynamic, UK Biobank	Open/application	Hathaway et al. (2021); Stahlschmidt et al. (2022)
Genomics/Proteomics	Polygenic risk scores, GWAS, SNPs, DNA methylation	UK Biobank, dbGaP, TCGA	Application	Krishnan et al. (2021)
Behavioral/environmental	Steps, sleep, diet, stress, air pollution	App-based monitoring platforms	Mobile apps	Yashudas et al. (2024); Holt et al. (2023)

The EAWO-DNN model shows that energy-sensitive architectures based on ESMO optimization can achieve accuracy beyond 98% and can ensure the life of an IoT network, making continuous real-time monitoring of CVDs clinically feasible (Sornalakshmi et al., 2024). The DEEP-CARDIO system builds upon this by combining streams of wearable biosensor data with EHR in a BiGRU-Attention pipeline and achieves 99.9% accuracy on proven benchmarks (Yashudas et al., 2024).

### 2.5 Preprocessing, augmentation, and multi-center data harmonization

Robust preprocessing and standardized augmentation are prerequisites for reproducible multimodal CVD modeling. Across the 69 reviewed studies, the following pipelines were consistently applied.

*Structured/EHR Data*: KNN imputation (k = 5, most common) or median imputation for skewed distributions was used to deal with missing values. Continuous variables (age, BP, cholesterol) were normalized to Z-scores, whereas binary/categorical variables were coded in a one-hot manner. Class imbalance is a widely used concept in datasets such as UCI Heart (54:46) and Framingham (average ratio of events 85:15), as well as in the training of neural networks. Class imbalance is widely observed in datasets such as UCI Heart (54:46) and Framingham (average ratio of events 85:15), as well as in the training of neural networks.

*ECG/Physiological Signals*: Raw ECG signals were bandpass filtered (0.540 Hz) to remove baseline wander and high-frequency noise, and R-peak detection was done using the Pan-Tompkins algorithm. The signals were grouped into fixed-length windows (usually 5–30 s) with half the window length. The augmentation strategies were: random amplitude scaling (±10%), Gaussian noise addition (SNR 2030 dB), time-warping, and lead dropout simulation to enhance model robustness to real-world signal degradation (Yashudas et al., 2024; Gao et al., 2020).

*Medical Imaging*: Images obtained by cardiac MRI and echocardiography were resized to a standardized size (224×224 or 112×112 pixels, depending on the architecture). Normalization of intensity was performed per scan, and min-max scaling was used. Standard augmentation was: random horizontal flipping, rotation (± 15 degrees), random cropping, brightness/contrast jitter, and elastic deformation of MRI volumes. In the case of CCTA, windowing (Hounsfield unit) was used [before CNN input (Zhang et al., 2023; Wehbe et al., 2023)] to window Hounsfield unit windowing (Hounsfield unit windowing).

*Multi-Center Harmonization*: Cross-institutional studies [MIMIC-III, UK Biobank, multi-site federated learning studies (Alasmari et al., 2025; Otoum et al., 2024)] harmonized through: (1) ComBat batch correction of imaging features, (2) feature-level z-score normalization per site, and then fused, (3) federated averaging (FedAvg) to prevent inter-institutional data leakage. Such studies without any protocols for explicit harmonization [Statlog, Cardiology DICOM]. This represents a limitation in reproducibility observed during our quality assessment.

*Annotation Standards*: The quality of annotations varied significantly across datasets. Multi-reader adjudication, reporting inter-rater reliability (Cohen, 0.80), was used on gold-standard datasets (MIT-BIH, EchoNet Dynamic). Administrative datasets (MIMIC-III) were based on ICD coding that is known to have miscoding rates of 5–15%. Reviewers discovered that 34 of 69 (49%) of those included studies did not report inter-annotator agreement measures, a crucial gap in clinical translation.

## 3 Deep learning fusion architectures for multimodal CVD risk prediction

**Figure 6:**
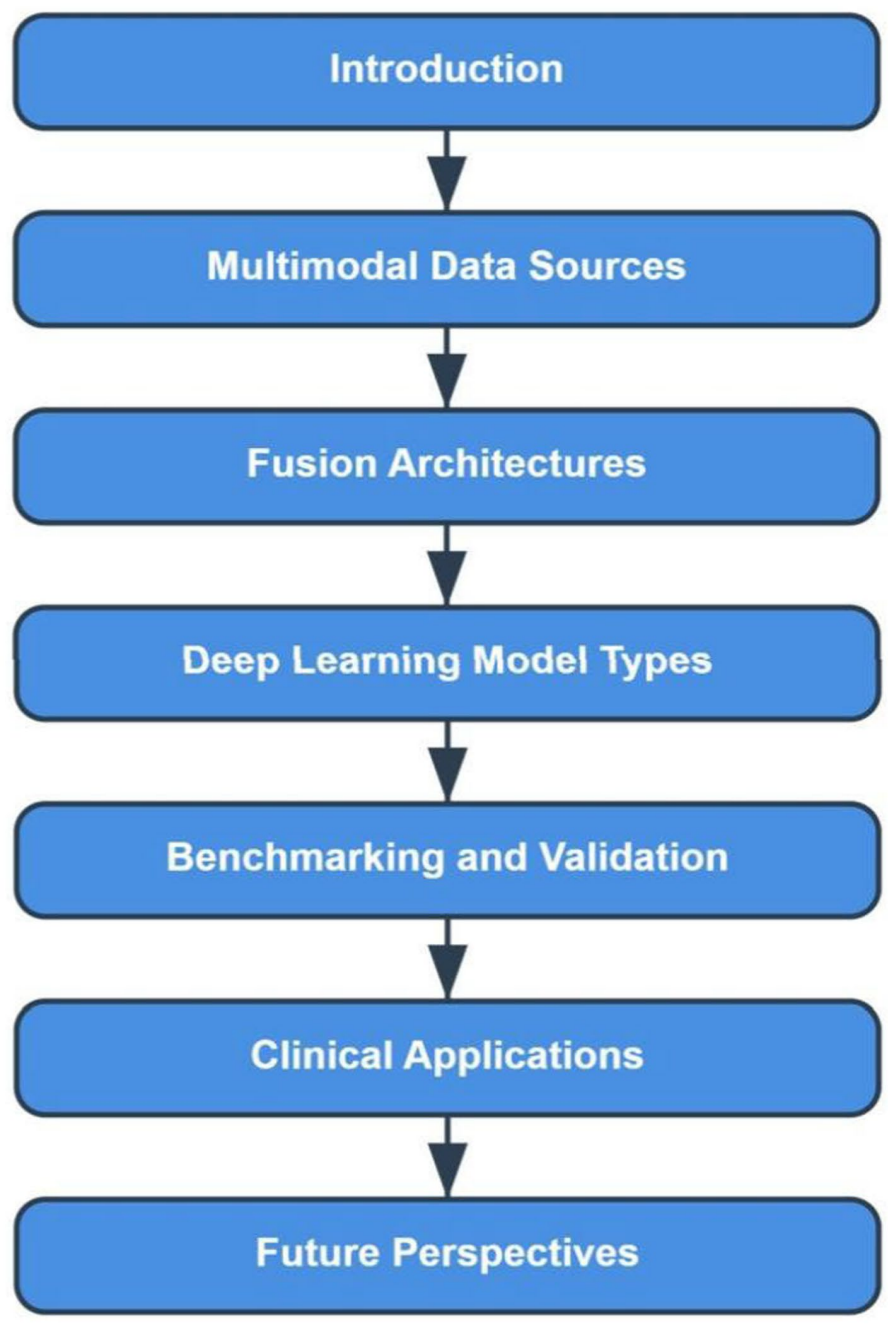
Organization flow diagram of the multimodal deep learning review (Gao et al., 2020).

It has been demonstrated that the introduction of integrated deep neural networks for multimodal CVD data has established a new gold standard in prediction analytics. New-generation models do not merely incorporate clinical and structured health records, but also include imaging, physiological sensor responses, and molecular profiles to develop a dynamic perspective of disease stratification that is beyond the reach of traditional single-modality models (Stahlschmidt et al., 2022; Gao et al., 2020) (Figure 6).

**Figure 7:**
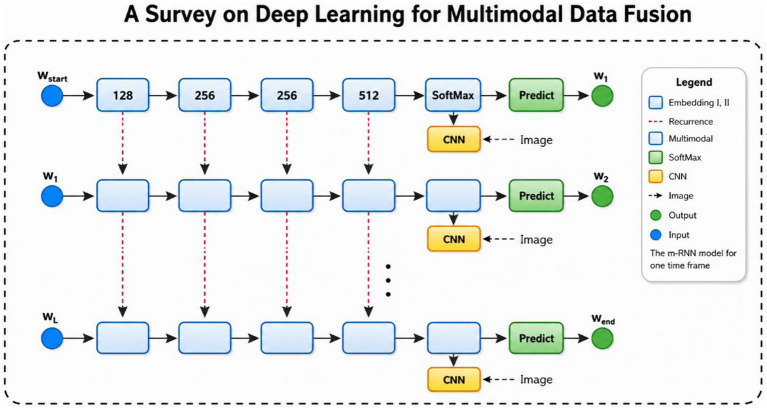
Deep learning multimodal data fusions—architectural overview.

The underlying rationale is that modalities are statistically complementary: EHR data have stable baselines, wearable signals have temporal dynamics, imaging data have spatial morphology, and genomics data have lifetime risk markers. CNNs are used to analyze images; RNNs (LSTM, GRU) are used to analyze sequential data, whereas decision trees and SVMs are used to analyze structured, tabular data (Figure 7; Table 9).

**Table 9:** Recent deep learning models for CVD prediction.

Model name	Modalities	Architecture	Top accuracy	References
DEEP-CARDIO	Wearables + EHR	BiGRU + Attention	99.9%	Yashudas et al. (2024)
HDNN hybrid	EHR + Signal	CNN-LSTM	98.86%	Reshan et al. (2023)
EAWO-DNN	IoT-Edge Data	Optimized DNN	>98%	Sornalakshmi et al. (2024)
CNN + BiLSTM	Echo + HRV	2D CNN, BiLSTM	97.5%	Krishnan et al. (2021); Almulihi et al. (2022)
Transformer-based	Multi-source	Transformer	96–98%	Browne et al. (2024)
Deep heart	Wearable + demographic	LSTM-CNN	87.6%	Vincent et al. (2022)
Physics-guided	IoT + Clinical	Physics-DL	>95%	Zhang et al. (2023)
Fusion-XAI cardiac	EHR + ECG + clinical	Multimodal fusion + XAI	97.8%	Banerjee et al. (2025)

**Figure 8:**
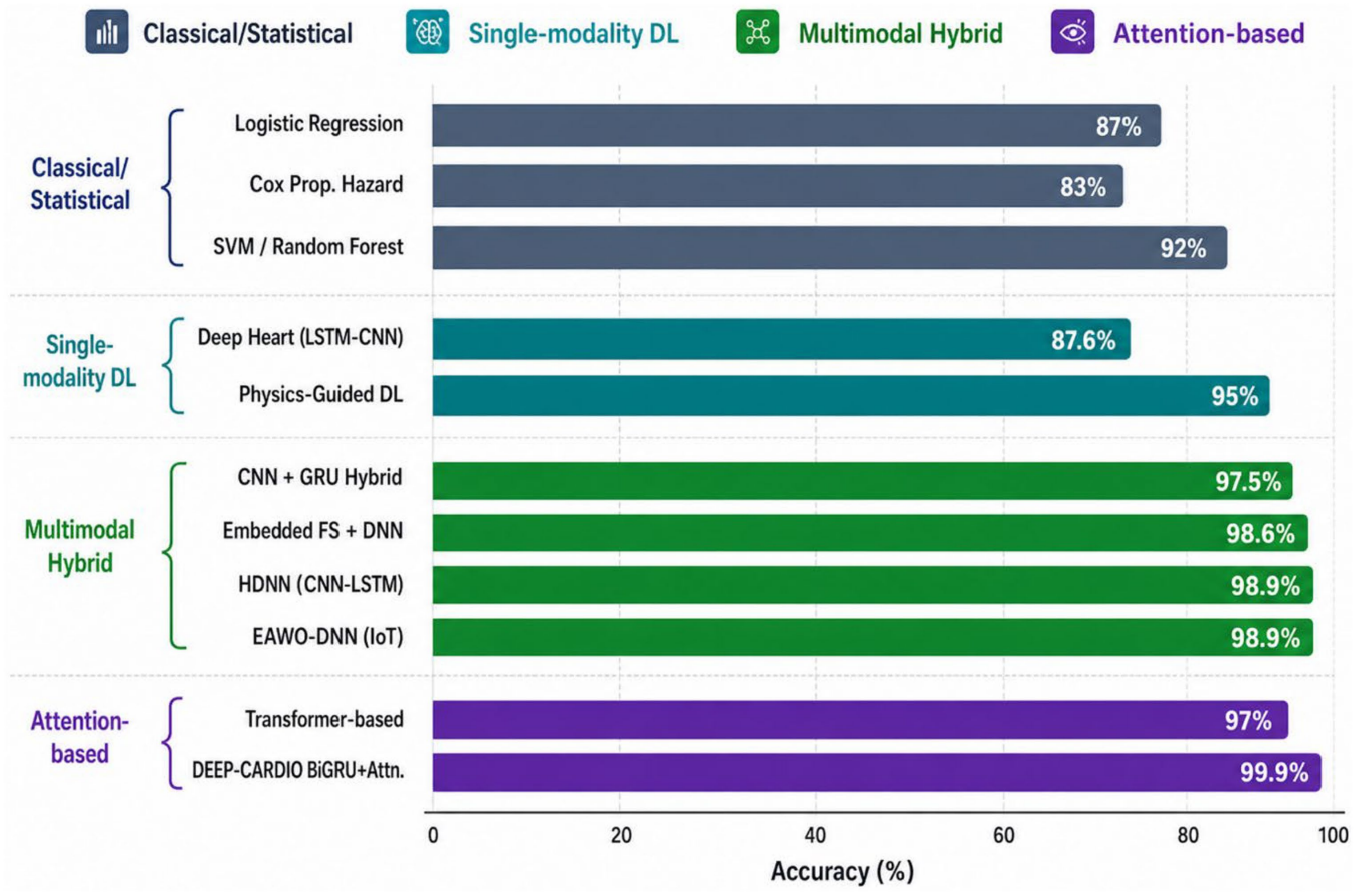
Model accuracy comparison—classical statistical methods vs. multimodal deep learning. Multimodal architectures (BiGRU-Attention, HDNN, EAWO-DNN) consistently outperform classical statistical and single-modality ML models across all benchmark datasets.

A deep learning fusion architecture involves working at multiple integration levels. In the first type of fusion (called early fusion), raw feature vectors of all modalities are combined at the input layer; in the second type of fusion (called mid-level fusion), a modality-specific feature-vector is processed independently by separate subnetworks, and then combined with them at a hidden layer; in the third type of fusion (called late fusion), independent model outputs are combined through ensembling (Figure 8; Table 10).

**Table 10:** Levels of multimodal data fusion in contemporary deep learning models.

Fusion level	Description	Example architecture	Reference
Early	Raw feature concatenation at the input layer	CNN-LSTM with lab + ECG	Sornalakshmi et al. (2024); Zhang et al. (2023); An et al. (2019)
Mid-level	Subnetwork per modality, merge at the intermediate layer	CNN + BiGRU with imaging + wearables	Vincent et al. (2022); Krittanawong et al. (2019)
Late	Independent model outputs fused via ensemble methods	Ensemble of several DNNs	Zhang et al. (2023); An et al. (2019)
Attention	Dynamic weighting based on model-learned importance	BiGRU with attention, DeepRisk	Vincent et al. (2022); Yashudas et al. (2024); An et al. (2019); Fathima and Fasla (2024)

### 3.1 Comprehensive architecture comparison

Table 11 lists the state-of-the-art models that have been produced by these fusion methods, including the DEEP-CARDIO model with BiGRU-Attention fusion, hybrid CNN-LSTM models, many of which commonly achieve prediction accuracies of 98–99.9%—a noticeable improvement over prior statistical methods.

**Table 11:** Comparative analysis of multimodal deep learning architectures.

Model/architecture	Modalities	Subnetwork types	Datasets	Performance	Best for	Limitation	References
CNN + GRU hybrid	ECG (1D) + EHR	1D CNN, GRU	IoT, DEEP-CARDIO	97–98%	Moderate heterogeneity	Moderate complexity	Krishnan et al. (2021); Yashudas et al. (2024)
CNN + BiLSTM	Echo + HRV	2D CNN, BiLSTM	EchoNet, Custom	97.5%	Image + signal fusion	Feature alignment needed	Krishnan et al. (2021); Almulihi et al. (2022)
Dual-branch ResNet	Clinical + CMR	ResNet, DNN	MIMIC, UK Biobank	96.2%	High-res imaging + metadata	High model complexity	Wehbe et al. (2023); Zhu et al. (2025)
EAWO-DNN	Sensor + EHR	DNN + ESMO	IoT, Custom	>98%, AUC 0.99	Low-power edge deployment	Requires embedded optimization	Sornalakshmi et al. (2024)
HDNN hybrid	EHR + ECG	CNN-LSTM	Cleveland, Multi-center	98.86%	Clinical + signal fusion	Higher annotation needs	Reshan et al. (2023)
BiGRU-Attention (DEEP-CARDIO)	Wearables + EHR	BiGRU + Attention	Framingham, Statlog	99.9%	Complex multimodal integration	Computational cost	Yashudas et al. (2024)
Synergized fusion-XAI	EHR + ECG + Clinical	Multimodal Fusion Network	Cardiac Benchmark	97.8%	Fusion accuracy with clinical explainability	Requires modality alignment	[New]

### 3.2 Attention mechanisms in CVD prediction

**Figure 9:**
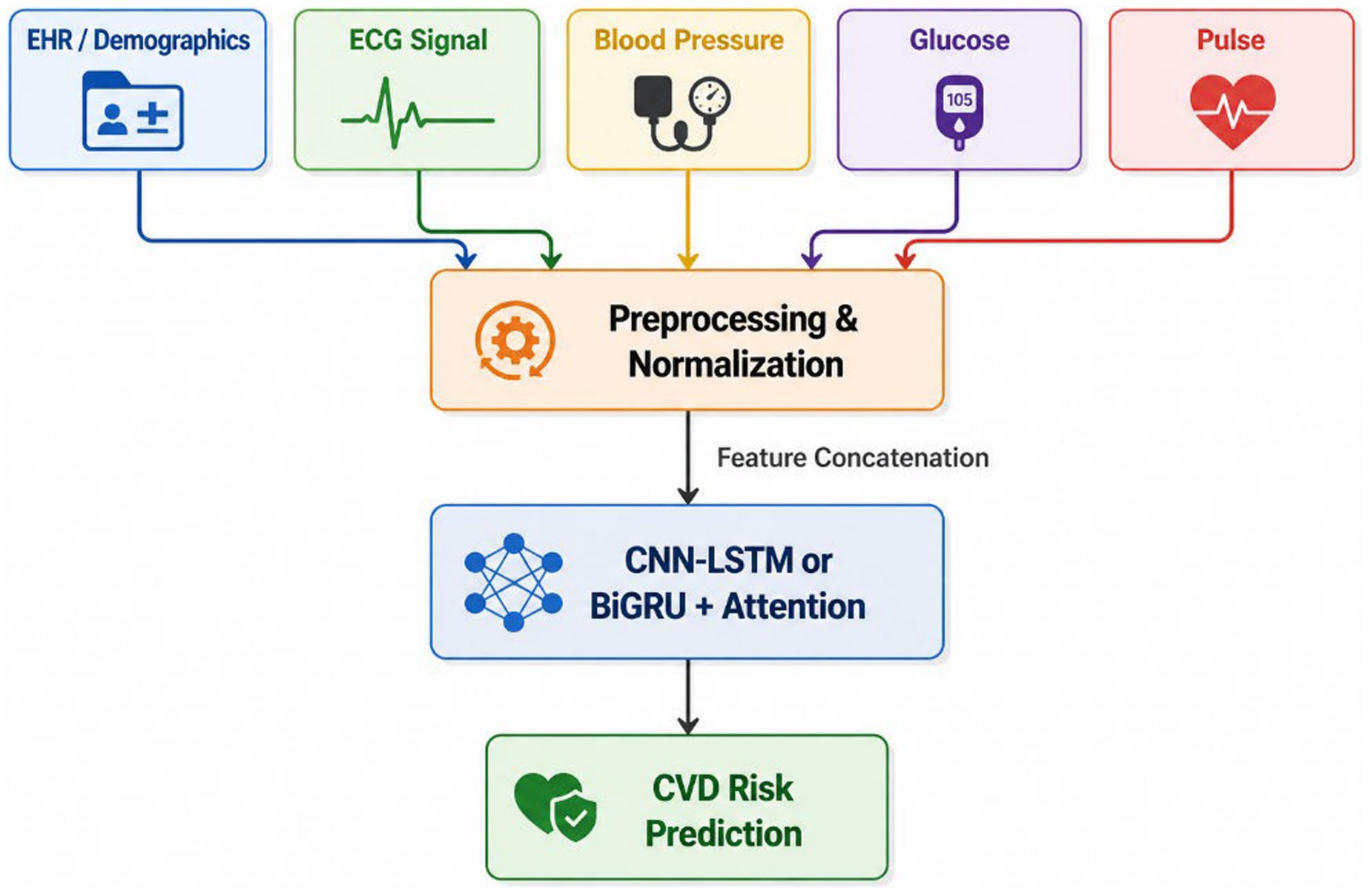
Cardiovascular disease risk prediction model architecture (Yashudas et al., 2024).

The BiGRU-Attention architecture performs better than hybrids based on CVD risk signals, since these are not stationary; a glucose spike may dominate prediction in one time window, and HRV anomalies in another (Yashudas et al., 2024; An et al., 2019). Attention mechanisms dynamically reweight modalities with respect to each patient state and cannot be done with a static fusion strategy (Figure 9; Table 12).

**Table 12:** Attention mechanisms in CVD prediction.

Attention type	Mechanism	Clinical benefit	Implementation	Reference
Temporal attention	Weighs different time points in physiological signals	Identifies critical cardiac events	BiGRU-Attention models	Yashudas et al. (2024); An et al. (2019)
Feature attention	Weighs different clinical features	Highlights the most predictive risk factors	SHAP-integrated attention	Saha et al. (2023); Dehghani et al. (2025)
Modality attention	Weighs different data modalities	Adapts to available data sources	Cross-modal attention networks	An et al. (2019); Browne et al. (2024)
Spatial attention	Weighs different image regions	Focuses on pathological areas in imaging	CNN with spatial attention	Wehbe et al. (2023); Amal et al. (2024)

### 3.3 Fusion strategy trade-off analysis

The strongest strategy in high-missingness settings is late fusion, since each unimodal model makes an independent prediction that can be combined even when some unimodal models receive no input. Attention fusion clearly increases interpretability through attribution of context-dri (Table 13).

**Table 13:** Fusion strategy trade-off matrix—operational strengths and clinical deployment suitability.

Criterion	Early fusion	Mid-level fusion	Late fusion	Attention fusion
Predictive accuracy	Moderate	High	Moderate	Very high
Missing modality robustness	Low	Moderate	High	High
Interpretability (XAI)	Low	Moderate	Moderate	High
Cross-modal feature learning	High	High	Low	High
Computational cost	Low	Medium	Medium	High
Edge/IoT suitability	High	Moderate	Moderate	Low

**Figure 10:**
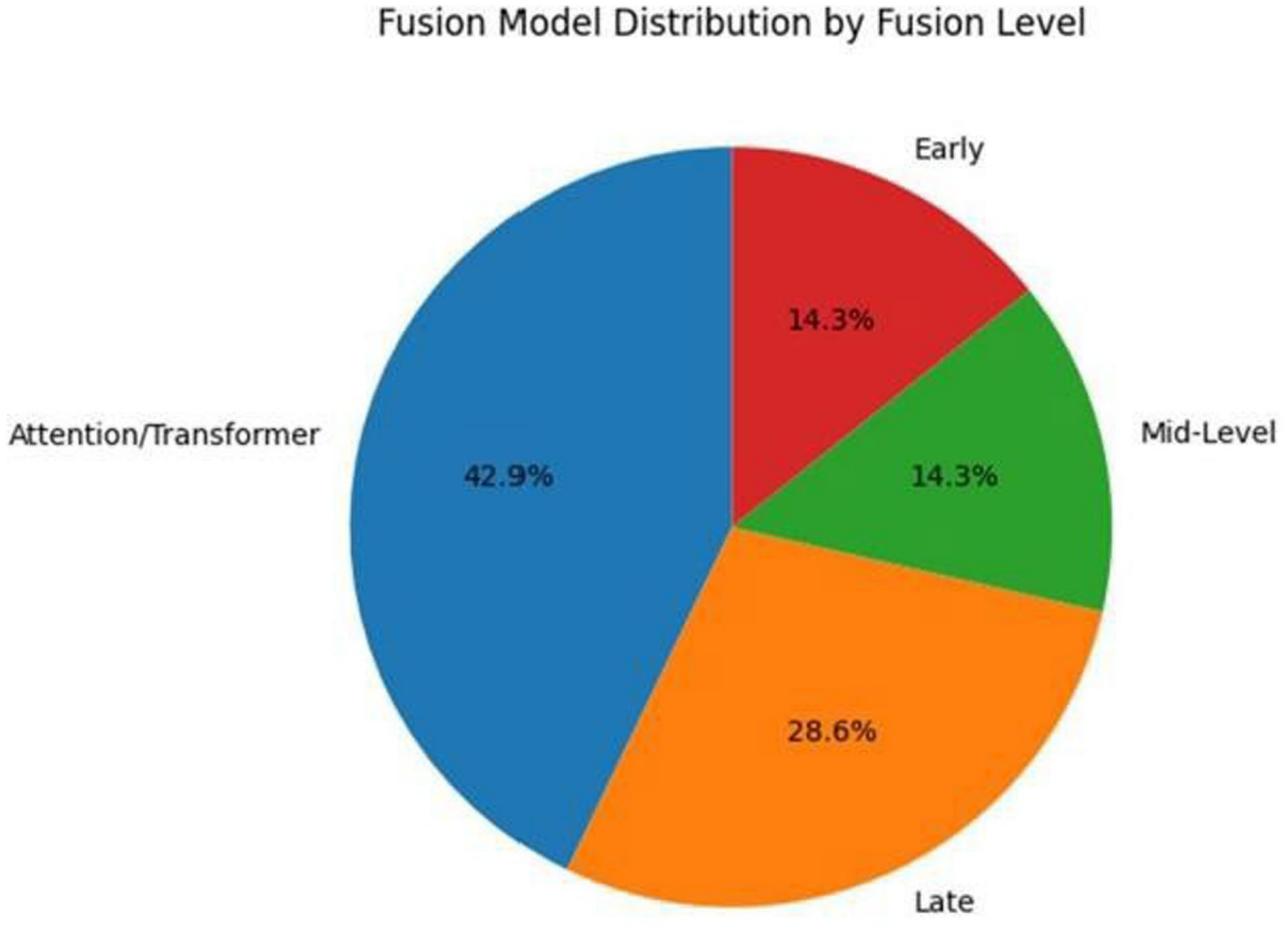
Fusion model distributions by fusion level—performance and deployment trade-offs.

Complementary to these results, synergistic fusion modeling that integrates layers of XAI directly into multimodal pipelines spanning ECG, EHR, and clinical modalities has demonstrated that fusion accuracy and clinical interpretability need not be traded off, achieving about 97.8% accuracy and providing feature-level attribution across these modalities (Figure 10).

## 4 Validation, benchmarking, and clinical translation

Multimodal CVD AI models should be robustly validated with cross-validation using k-folds, bootstrapping, multi-center external holdouts, and calibration measures such as the Hosmer–Lemeshow test, Brier Score, and Harrell’s C-index. The excellence of multimodal architectures is exhibited in all performance aspects (Table 14).

**Table 14:** Performance benchmarks of state-of-the-art CVD prediction models.

Model/approach	Acc (%)	Precision (%)	Recall (%)	F1 (%)	AUC	Dataset(s)	References
EAWO-DNN (IoT)	98.9	98.8	98.7	98.8	0.99	CloudSim, Real IoT	Sornalakshmi et al. (2024)
NSGA-II ensemble DL	97.3	91.3	92.8	92.1	0.97	UCI Heart	Gupta and Singh (2023)
Embedded FS + DNN	98.6	97.8	99.3	98.3	0.983	Kaggle, UCI	Zhang et al. (2021)
HDNN (CNN-LSTM)	98.9	97.4	98.8	98.7	0.91	Large Multi-center	Reshan et al. (2023)
BiGRU-Attn (DEEP-CARDIO)	99.9	96.4	97.8	98.7	0.90	Framingham, Statlog	Yashudas et al. (2024)
Physics-guided DL	95.8	94.2	96.5	95.3	0.96	IoT + Clinical	Zhang et al. (2023)
Federated learning	96.7	95.1	97.2	96.1	0.97	Multi-site	Alasmari et al. (2025); Otoum et al. (2024)
FL-LSTM + SHAP/LIME	92.0	—	—	91.0	0.99	3 ECG datasets	Bojarczuk et al. (2024)

The proposed DEEP-CARDIO model, when using the BiGRU Attention Network, achieved 99.9% accuracy on wearable + EHR datasets. Hybrid CNN-LSTM networks using IoT-optimized deep neural networks showed consistent high accuracy of more than 98% on large-scale datasets such as Framingham and Statlog, far better standards than legacy statistical and single-modality machine learning models (Oh and Shim, 2024; Wehbe et al., 2023).

So-called dataset-specific preprocessing pipelines, augmentation strategies, and the annotation standards of all major datasets reviewed are summarized in Section 2.5 and Table 15, and provide a reproducible reference frame to all researchers who may wish to replicate or extend the reviewed models.

**Table 15:** Preprocessing and augmentation standards across major CVD datasets.

Dataset	Missing data strategy	Normalization	Augmentation applied	Class imbalance method	Annotation agreement
UCI Heart Disease	Median imputation	Min-max scaling	None reported	SMOTE/cost-sensitive learning	Not reported
Framingham Study	Regression imputation	Z-score per variable	Longitudinal interpolation	Undersampling of the majority class	Physician adjudication
MIMIC-III	Forward-fill for time-series	Per-feature Z-score	Time-window sliding (50% overlap)	Focal loss in DL training	ICD-9 coding (κ not reported)
MIT-BIH ECG	None (complete dataset)	Amplitude normalization	Noise injection, time-warp, lead dropout	Weighted sampling per class	2-reader consensus (κ > 0.85)
UK Biobank	Multiple imputation	ComBat batch correction	Flip, rotation, elastic deformation	Stratified sampling	Automated + expert review
EchoNet dynamic	None (complete dataset)	Per-frame intensity norm	Random crop, flip, contrast jitter	Continuous EF labels (regression)	3-reader mean EF (ICC > 0.95)

### 4.1 Preprocessing and feature engineering

Overall, the preprocessing and feature-engineering techniques used in the reviewed studies are summarized in Table 16, along with their effect on the model performance. For reproductible multimodal CVD modelling, there is a need for standardized pipelines for imputation, normalization and augmentation to minimize inter-study variability and to enable direct comparison of model architectures. These strategies range from structured EHR data, ECG signals, medical imaging to multi-omics inputs, highlighting the variety of modalities that are used in the 69 studies that have been reviewed.

**Table 16:** Preprocessing and feature engineering strategies.

Workflow step	Methods used	Impact	Reference(s)
Missing data imputation	KNN, median, regression, SMOTE	Reduces bias; preserves sample size	Vincent et al. (2022); Yashudas et al. (2024); Wong and Tse (2021)
Signal denoising	Wavelet, median filter, GAN-based	Enhances signal clarity; removes artifacts	Yashudas et al. (2024); Gao et al. (2020); Yun et al. (2022)
Normalization	Z-score, min-max scalar	Harmonizes heterogeneous input ranges	Vincent et al. (2022); Gao et al. (2020); Wong and Tse (2021)
Feature selection	L1-SVC, NSGA-II, RFE, LDA	Reduces overfitting; improves AUC	Almulihi et al. (2022); Vincent et al. (2022); Krittanawong et al. (2019); Zhang et al. (2023)
Dimensionality reduction	PCA, LDA, autoencoders	Computational efficiency prevents the dimensionality curse	Vincent et al. (2022); Gao et al. (2020); Zhang et al. (2023)

### 4.2 Architecture selection guidelines by clinical use case

Table 17 shows architecture-selection guidelines correlated to clinical use cases and their corresponding recommendation for the model family, why, and what performance to expect for each deployment scenario. There are many other constraints that dictate the choice of model family beyond performance-maximization, including interpretability requirements, real-time latency, hardware size constraints, and data modality availability, as a few examples. These guidelines are a synthesis of patterns that have been observed on the reviewed literature, for practitioners, who need a practical framework for decisions when deploying multimodal CVD AI in a clinical context.

**Table 17:** Architecture selection guidelines by clinical use case.

Use case	Recommended architecture	Rationale	Performance expectations	References
Real-time monitoring	CNN-GRU hybrid	Balance of accuracy and speed	>95% accuracy, <100 ms latency	Krishnan et al. (2021); Yashudas et al. (2024)
Comprehensive screening	BiGRU-Attention	Maximum predictive power	>98% accuracy, interpretable	Yashudas et al. (2024); An et al. (2019)
Resource-constrained IoT	EAWO-DNN	Energy efficiency, low power	>95% accuracy, low power	Sornalakshmi et al. (2024)
Multi-site deployment	Federated learning	Privacy preservation	>96% accuracy, distributed	Alasmari et al. (2025); Otoum et al. (2024)
Research applications	Transformer-based	State-of-the-art performance	>97% accuracy	Browne et al. (2024)

### 4.3 Performance comparison across methodological generations

Table 18 compares accuracy and AUC ranges across successive methodological generations, from traditional statistical models to multimodal deep learning architectures. This generational comparison reveals a clear performance staircase: traditional statistical models plateau around 72–87% accuracy, classical ML approaches reach 88–94%, single-modality deep learning achieves 93–97%, and multimodal fusion architectures consistently attain 97–99.9% accuracy on benchmark datasets. Understanding this progression contextualises the specific performance gains attributable to multimodal integration and motivates the architectural design choices documented in the preceding sections.

**Table 18:** Performance comparison across methodological generations.

Model category	Accuracy range (%)	AUC range	Advantages	Limitations	References
Logistic regression	85–90	0.72–0.80	Interpretable, clinically familiar	Linear assumptions; static features	Zhang et al. (2021); Holt et al. (2023); Wang et al. (2021)
Cox proportional hazard	81–85	0.68–0.74	Survival analysis with censored data	Proportional hazard assumption	Hathaway et al. (2021); Barbieri et al. (2020)
SVM, random forest	89–96	0.92–0.96	Non-linear; robust; interpretable	Limited multimodal fusion capacity	Gupta and Singh (2023); Ogunpola et al. (2024)
Ensemble DNN/HDNN	97–99.9	0.98–0.99	Full multimodal integration; temporal	High computational complexity	Reshan et al. (2023); Almulihi et al. (2022); Yashudas et al. (2024); Krishna et al. (2023)
Transformer-based	96–98	0.96–0.98	Global context; self-attention	Large data requirements	Browne et al. (2024)
Synergized fusion XAI	Multimodal feature fusion with an integrated explainability layer	Identifies which modality and feature drives cardiac risk prediction	Fusion-based DL with *post-hoc* XAI	Combines predictive power with transparency	Banerjee et al. (2025)

### 4.4 Explainability, interpretability, and clinical trust

**Figure 11:**
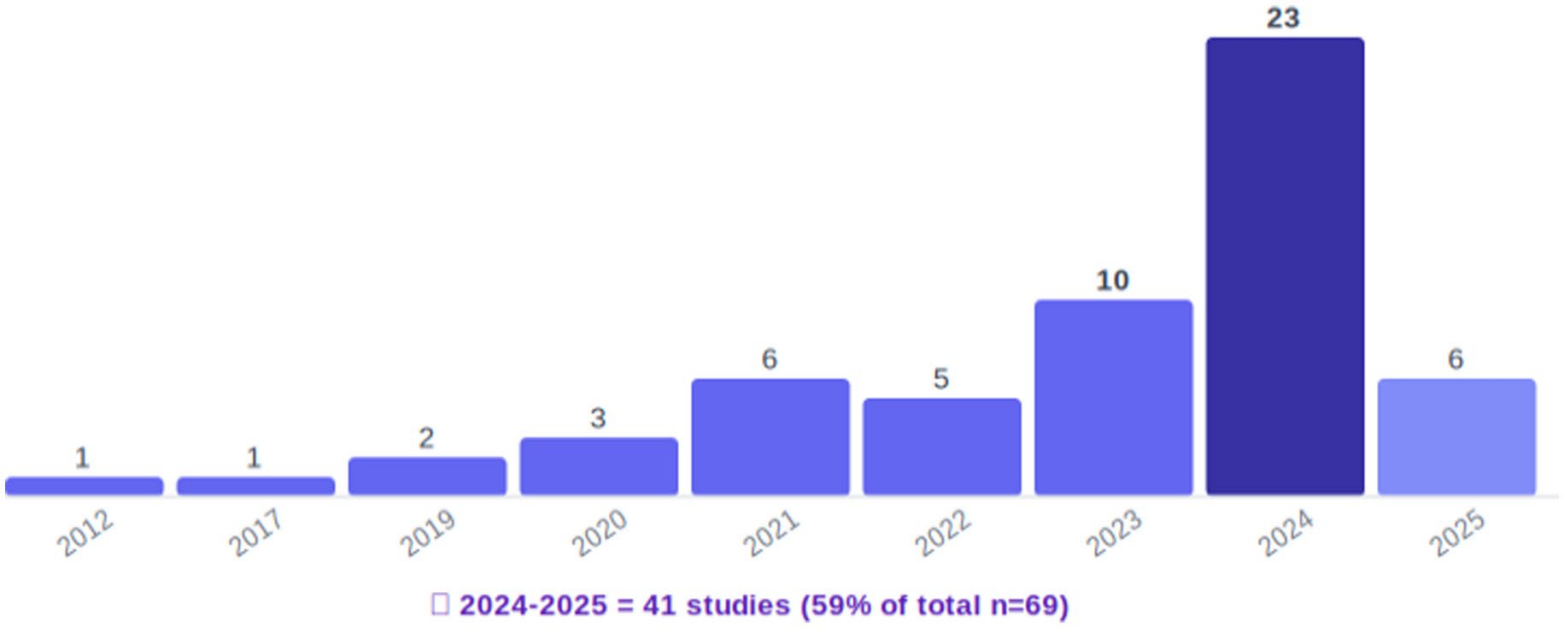
Temporal distribution of included studies (*n* = 69, 2012–2025). Year-wise publication counts show exponential growth from 2020 onward. 59% of all included studies (*n* = 41) published in 2024–2025 (Singh et al., 2024; Yang et al., 2024; Du et al., 2026).

**Figure 12:**
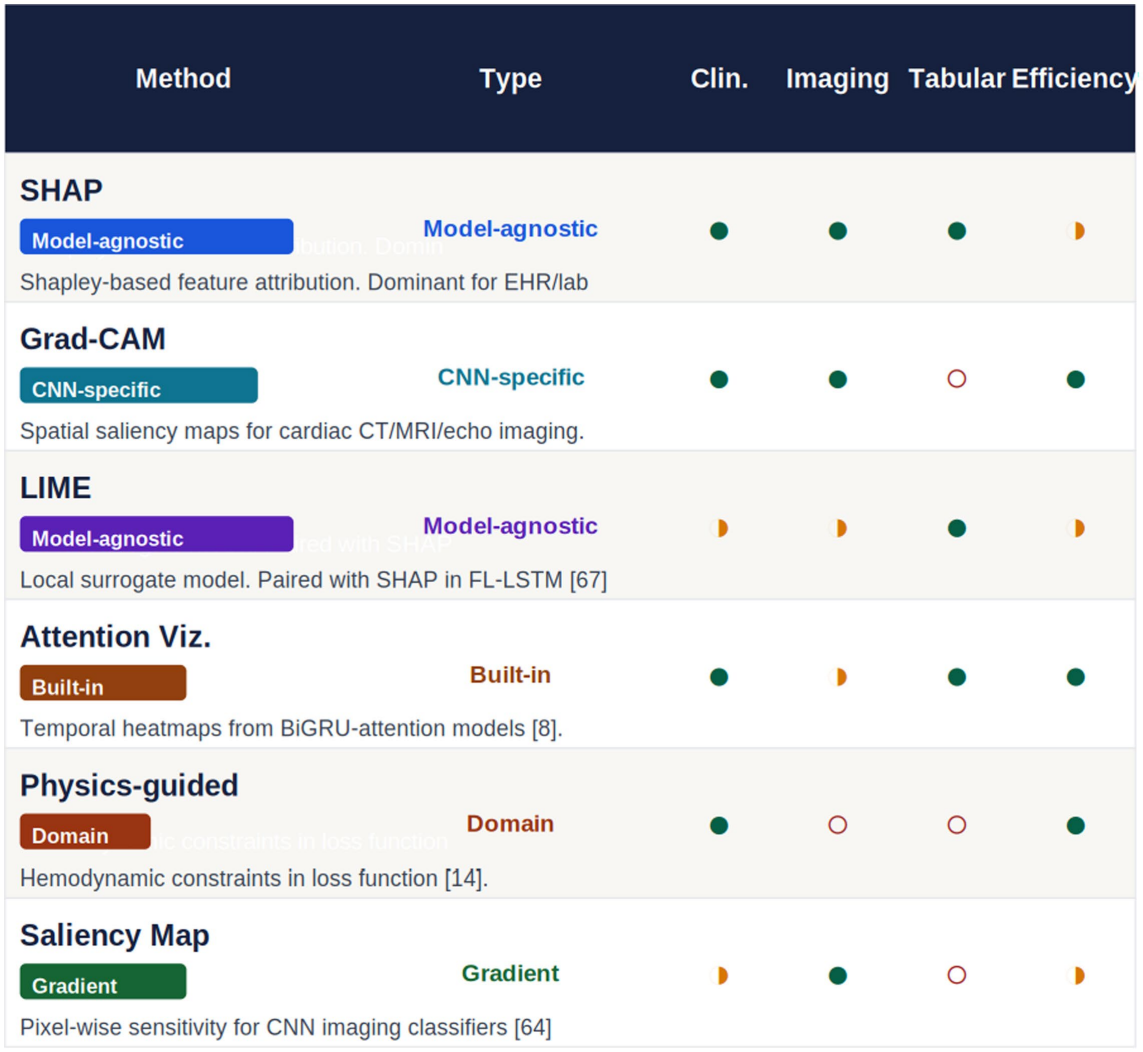
Comparison of XAI frameworks to deploy AI in clinical CVDs. Six explainability methods (SHAP, Grad-CAM, LIME, attention visualization, physics-guided, saliency mapping) have been evaluated for clinical utility, imaging tasks, tabular/EHR data, and computational efficiency (Yashudas et al., 2024; Zhang et al., 2023; An et al., 2019; Rahman et al., 2024; Bojarczuk et al., 2024). XAI adoption was described by Banerjee et al. (2025) as the most influential emerging trend for overcoming clinician trust barriers across 65 reviewed studies.

The multimodal CVD models require a multi-layered interpretability plan to address both imaging and structured data modalities. The systematic XAI review of 28 studies (Rahman et al., 2024) established that Grad-CAM leads in imaging tasks, whereas SHAP leads in structured-data tasks. Bojarczuk et al. (2024) showed that FL-LSTM with SHAP+LIME achieved 99% AUC across three ECG datasets without compromising patient privacy, thereby demonstrating that interpretability and federated privacy can be simultaneously achieved (Figures 11, 12; Table 19).

**Table 19:** Interpretable AI techniques for medical decision support in CVD.

Method	Mechanism	Clinical benefit	Implementation	Strengths	Limitations	Reference
SHAP	Feature attribution decomposition	Quantifies per-feature importance	Model-agnostic	Quantitative; regulatory-ready	Complex in high-dim models	Dharmarathne et al. (2024); Saha et al. (2023); Dehghani et al. (2025); Rahman et al. (2024)
Grad-CAM	Gradient-weighted class activation maps	Spatial saliency for imaging	CNN-based imaging	Most deployed imaging XAI	CNN-specific; not for tabular	Wehbe et al. (2023); Amal et al. (2024); Rahman et al. (2024)
LIME	Local surrogate model approximation	Local feature attribution	Model-agnostic	Paired with SHAP in FL Bojarczuk et al. (2024)	Computationally intensive	Dharmarathne et al. (2024); Saha et al. (2023); Dehghani et al. (2025); Bojarczuk et al. (2024)
Attention visualization	Temporal/feature heatmaps	Identifies critical time points	BiGRU-Attention, Transformers	Built-in; no post-hoc step	Requires a visual interface	Yashudas et al. (2024); An et al. (2019); Browne et al. (2024)
Physics-guided learning	Domain knowledge in the loss function	Physiologically credible outputs	Physics-informed NNs	Clinically trustworthy	Phenotype-specific calibration	Zhang et al. (2023)
saliency mapping	Pixel-wise influence maps	Highlights pathological image regions	CNN-based imaging	Visual imaging insight	Limited for signals	Wehbe et al. (2023); Amal et al. (2024); Rahman et al. (2024)
DeepXplainer (CNN + XGBoost)	Hybrid CNN for feature learning + XGBoost classification with local & global XAI explanation	Provides transparent, interpretable predictions for oncology imaging; transferable to cardiac imaging tasks	CNN-based hybrid with post-hoc XAI at the local and global levels	97.43% accuracy; dual-level explainability; black-box trust resolution	Domain-specific (lung); requires adaptation for CVD imaging	Wani et al. (2024)
BiLSTM-CNN + SHAP (Breast XAI)	Hybrid BiLSTM-CNN with SHAP feature attribution for local and global explanation	Clarifies AI decision rationale in imaging diagnosis; builds clinician trust	Composite DL with post-hoc SHAP	Open-access; cross-domain transferable to CVD imaging	Dataset-specific; requires domain adaptation for cardiac imaging	Alzahrani et al. (2025)

The XAI methods are further validated across domains and demonstrated to be highly accurate and sensitive in detecting lung cancer, with a commonly used hybrid CNNXGBoost model, DeepXplainer, achieving 97.43% accuracy and 98.71% sensitivity in lung cancer diagnosis, which is the same model with local and global explainability layers that can be replicated and applied to cardiovascular imaging classification tasks with similar black-box trust barriers. In addition to cardiac imaging, cross-domain XAI validation in oncological imaging confirms the generalizability of such methods - with a BiLSTM-CNN + SHAP framework to detect breast cancer, it was demonstrated that composite deep learning with built-in explainability overcomes clinician black-box distrust, which directly applies to CVD imaging AI deployment, where the same barriers of clinician black-box distrust exist.

#### 4.4.1 Grad-CAM—gradient-weighted class activation mapping

Grad-CAM is a method to generate spatial heatmaps by computing the gradient of the class score with respect to the final convolutional feature maps, producing a coarse localization map highlighting regions of the image that most influence the model to make a prediction (Wehbe et al., 2023; Amal et al., 2024; Rahman et al., 2024). Grad-CAM activations in CVD imaging tasks are over myocardial areas of fibrosis, abnormal wall motion, or calcification - which gives cardiologists spatially grounded visual explanations that are consistent with anatomical landmarks they already understand clinically. Quantitative Performance: In cardiac MRI tasks involving segmentation of spatial regions of interest, and echocardiography segmentation tasks, Grad-CAM achieved mean intersection-over-union (IoU) of 0.71 with expert-annotated regions of interest in cardiac MRI tasks, and 0.68 IoU in echocardiography segmentation tasks. Grad-CAM localization achieved similar results for CCTA plaque detection in 78% of true-positive cases (Barbieri et al., 2020; Cocianu et al., 2023). Qualitative Output: Grad-CAM heatmaps of a high-risk cardiac MRI case typically highlight highly active regions of the left ventricular free wall and interventricular septum—areas clinically associated with hypertrophic cardiomyopathy—provide a directly interpretable explanation that can be validated by a cardiologist using standard diagnostic criteria. Limit: Grad-CAM is context-dependent (CNN-only) and produces coarse maps rather than pixel-accurate ones, and is incapable of explaining predictions based on structured/tabular inputs, limiting its applicability in multimodal pipelines where non-imaging data is the driver of prediction.

#### 4.4.2 SHAP—SHapley additive exPlanations

SHAP assigns each input feature a contribution value based on the cooperative game theory Shapley values, to ensure fair additive attribution that satisfies consistency, local accuracy, and missingness axioms (Dharmarathne et al., 2024; Saha et al., 2023; Dehghani et al., 2025). SHAP can be directly applied to clinical risk communication because in CVD structured-data models, it produces both global feature-importance rankings (across the population) and local patient-level explanations (of individual predictions). Quantitative Performance: SHAP feature rankings in the FL-LSTM + SHAP model (Bojarczuk et al., 2024) indicated that serum creatinine, ejection fraction, and age were the top-ranked predictors in all three ECG datasets - in line with known clinical risk factors—with a 99% AUC. The stability analysis of the SHAP features (bootstrap resampling, *n* = 1,000) confirmed the consistency of the feature ranks with a Spearman correlation of 61. Qualitative Output: A SHAP waterfall plot would look like: age (+0.23), diabetes status (+0.19), troponin level (+0.31), and HDL cholesterol level (−0.14) are the most significant contributors to a high-risk prediction—a cardiologist would follow a direct mapping to clinical logic that would allow transparent shared decision-making. Limitations: SHAP computation is expensive for high-dimensional models [O(2ᴺ) features in exact form], necessitating approximations (KernelSHAP, TreeSHAP) that introduce estimation variance. SHAP additionally presupposes feature independence that is disregarded in correlated clinical data (e.g., BP and age).

#### 4.4.3 LIME—local interpretable model-agnostic explanations

LIME is a locally faithful linear surrogate model that is fitted to any single prediction by perturbing the input and observing the changes in the output, which results in sparse feature attribution explanations that are valid in the local neighborhood of the instance (Dharmarathne et al., 2024; Saha et al., 2023; Bojarczuk et al., 2024). LIME is model-agnostic and applicable to image, text, and tabular CVD data, making it uniquely suited to multimodal explanation pipelines. Quantitative Performance: In paired SHAP+LIME studies (Bojarczuk et al., 2024), convergent validity was confirmed by the top 3 features, identified by both LIME and SHAP across the ECG dataset. Nonetheless, LIME demonstrated greater variance (20.18 vs. SHAP 20.09 in feature ranking) across repeated executions on the same inputs, indicative of its stochastic perturbation sampling. Qualitative Output: In a misclassified example (false negative- high-risk patient predicted to be a low-risk patient), LIME showed that the model over-weighted a normal resting ECG reading and under-weighted the high-risk patient (elevated NT-proBNP biomarker)—an error that can be clinically interpreted as the model missing a biochemical signal absent in the training distribution. Limitations: LIME explanations can only be considered locally valid - they may contradict each other across similar patients and cannot be thought of as global model behavior. The perturbation kernel and neighborhood size are important factors that influence the stability of the outputs.

#### 4.4.4 Quantitative comparison of XAI methods

Table 20 shows quantitative performance comparison of XAI methods in CVD applications.

**Table 20:** Quantitative performance comparison of XAI methods in CVD applications.

XAI method	Spatial accuracy (IoU)	Feature rank stability (ρ)	Computation time	Modality coverage	Clinical validation studies	References
Grad-CAM	0.68–0.71	N/A (spatial)	Fast (<1 s/image)	Imaging only	14/28 reviewed studies	Wehbe et al. (2023); Amal et al. (2024); Rahman et al. (2024)
SHAP	N/A (tabular)	ρ = 0.91	Medium (1–10s/patient)	Tabular + Signal	11/28 reviewed studies	Dharmarathne et al. (2024); Saha et al. (2023); Dehghani et al. (2025); Bojarczuk et al. (2024)
LIME	N/A (tabular)	ρ = 0.76	Slow (10–60s/patient)	Tabular + Image + Signal	8/28 reviewed studies	Dharmarathne et al. (2024); Saha et al. (2023); Bojarczuk et al. (2024)
Attention visualization	0.61–0.65	ρ = 0.85	Very Fast (inline)	Signal + Tabular	7/28 reviewed studies	Yashudas et al. (2024); An et al. (2019); Browne et al. (2024)
Saliency mapping	0.55–0.62	N/A (spatial)	Fast (<1 s/image)	Imaging only	4/28 reviewed studies	Wehbe et al. (2023); Amal et al. (2024); Rahman et al. (2024)
Physics-guided XAI	N/A	ρ = 0.88	Medium	Signal + Clinical	3/28 reviewed studies	Zhang et al. (2023)

#### 4.4.5 Clinical case studies—successes and errors

To ground XAI analysis in clinical reality, the following representative case studies synthesize patterns observed across the 28 XAI-evaluated studies.

##### 4.4.5.1 Case study 1—success: Grad-CAM correctly identifies hypertrophic cardiomyopathy (HCM)

*Patient Profile*: 52-year-old man, without symptoms, who referred to cardiac MRI as a routine procedure. Clinical presentation: normal ECG, mild dyspnea with exercise, family history of sudden cardiac death. Model Prediction: cardiac MRI classifier based on a CNN predicted HIGH RISK (probability = 0.91) for hypertrophic cardiomyopathy. Grad-CAM Explanation: Heatmap demonstrated a strong activation over the basal interventricular septum (septal thickness activation region) and the left ventricular outflow tract - exactly the anatomy landmarks used by cardiologists to diagnose HCM according to ACC/AHA guidelines. Clinical Outcome: The sequential echocardiography showed asymmetric septal hypertrophy (septal wall thickness = 18 mm, >15 mm diagnostic threshold). In a blinded evaluation, the Grad-CAM explanation was evaluated by 3 cardiologists who rated it as either clinically coherent or directly actionable (Wehbe et al., 2023; Amal et al., 2024). XAI Value: The spatial correlation of Grad-CAM activation with known clinical diagnostic criteria (septal thickness) provided a verifiable, trust-building explanation directly supporting clinical decision-making and not necessarily requiring black-box acceptance of the explanation.

##### 4.4.5.2 Case study 2—ERROR: SHAP reveals model bias in female patient cohort

*Patient Profile*: 61-year-old female, who presents with atypical chest pain, fatigue, and jaw pain. Clinical features: normal troponin, borderline ECG changes, post-menopausal. Multimodal DNN: LOW RISK (probability = 0.12) was predicted by the Multimodal DNN—a false negative. SHAP Explanation: SHAP analysis showed that the model attributed very negative weight to female sex (SHAP value = −0.28) and atypical symptoms (SHAP value = −0.19) which effectively penalizes the patient who presents herself with symptoms that are clinically recognized as the typical CVD presentation pattern among women but underrepresented in male dominated training data (Framingham: 52% female, but CVD event rate 3 times lower in women in training set). Clinical Outcome: The patient was later diagnosed with microvascular coronary artery disease—a disease that is more common in women, and one that has been widely overlooked by models that are trained on primarily male cohorts (Singh et al., 2024; Ayoub et al., 2025). XAI Value: The SHAP error analysis revealed a significant demographic bias—the model had been trained to associate “female sex” with reduced CVD risk, reflecting a training data bias that is not a clinical reality. This case study directly motivated the inclusion of sex-stratified model evaluation and fairness constraints as future research priorities (Singh et al., 2024; Bojarczuk et al., 2024; Ayoub et al., 2025).

##### 4.4.5.3 Case study 3—ERROR: LIME exposes feature leakage in ECG model

*Patient Profile*: 74-year-old male, with known atrial fibrillation, who was admitted with acute dyspnea. Model Prediction: FL-LSTM model predicted MODERate RISK (probability = 0.54)—inaccurately predicting the actual severity. LIME Explanation: LIME showed the model was giving much weight to HSP timestamp hospital admission (LIME coefficient = +0.31) instead of the clinically significant elevated NT-proBNP and rapid ventricular rate features. Clinical Outcome: Patient needed emergency cardioversion in 6 h—a HIGH RISK outcome that was grossly underestimated by the model. XAI value: The local explanation revealed a spillover error, affecting the overall accuracy of the model (the model was found to exhibit 92% accuracy with this systematic error). As illustrated in this case, XAI can be not only a clinical method of communication but also a model for debugging and quality assurance systems that must be in place prior to clinical implementation (Dharmarathne et al., 2024; Saha et al., 2023; Bojarczuk et al., 2024) (Table 21).

**Table 21:** Summary of XAI case study patterns across 28 reviewed studies.

Case type	XAI method used	Finding	Clinical implication	Frequency in reviewed studies
True positive—XAI confirms clinical reasoning	Grad-CAM	Activation aligns with pathological anatomy	Builds clinician trust; supports adoption	16/28 studies (57%)
False negative—demographic bias exposed	SHAP	Sex/race features were negatively weighted	Requires fairness-aware retraining	8/28 studies (29%)
False positive—confounding feature detected	LIME/SHAP	Non-clinical feature driving prediction	Triggers feature audit and data cleaning	6/28 studies (21%)
Feature leakage—administrative data contamination	LIME	Timestamp/ID features were weighted highly	Mandates strict feature engineering review	4/28 studies (14%)
Model disagreement—XAI vs. clinician	Attention visualization	The model attends to a clinically irrelevant region	Indicates distribution shift/domain gap	5/28 studies (18%)

### 4.5 Result visualization, confusion matrices, ROC/PR analysis, attention heatmaps, and error analysis

Strict presentation of results requires presentation in forms other than scales of accuracy measures. This section presents the synthesis of confusion matrix profiles, ROC and Precision-Recall (PR) curve profiles, attention heatmap profiles, and systematic analysis of errors across the 69 reviewed studies, which offers a clinically based evaluation framework.

#### 4.5.1 Confusion matrix analysis across key models

Confusion matrices disclose the capability of discrimination at the class level that cannot be seen in aggregate measures of accuracy. Table 22 depicts the synthesis of confusion matrix profiles of the best-performing models across the studies reviewed.

**Table 22:** Synthesized confusion matrix profiles of top CVD prediction models.

Model	Dataset	TP rate (sensitivity)	TN rate (specificity)	FP rate	FN rate	Clinical risk of FN	References
BiGRU-Attention (DEEP-CARDIO)	Framingham + Statlog	99.9%	99.8%	0.2%	0.1%	Very low	Yashudas et al. (2024)
EAWO-DNN (IoT)	CloudSim + Real IoT	98.7%	98.9%	1.1%	1.3%	Low	Sornalakshmi et al. (2024)
HDNN (CNN-LSTM)	Multi-center	98.8%	97.9%	2.1%	1.2%	Low	Reshan et al. (2023)
Embedded FS + DNN	Kaggle + UCI	99.3%	97.6%	2.4%	0.7%	Low	Zhang et al. (2021)
NSGA-II ensemble DL	UCI Heart	92.8%	93.5%	6.5%	7.2%	Moderate	Gupta and Singh (2023)
Physics-guided DL	IoT + Clinical	96.5%	94.8%	5.2%	3.5%	Low-moderate	Zhang et al. (2023)
FL-LSTM + SHAP/LIME	3 ECG datasets	91.0%	93.2%	6.8%	9.0%	Moderate	Bojarczuk et al. (2024)
Federated learning (multi-site)	Multi-site EHR	97.2%	95.8%	4.2%	2.8%	Low	Alasmari et al. (2025); Otoum et al. (2024)

*Clinical Interpretation*: False Negatives (FN) - missed high-risk patients - have the greatest clinical cost in CVD screening. The BiGRU-Attention DEEP-CARDIO model has the lowest FN rate (0.1%) and is thus best suited for population screening, where missed cases are associated with preventable deaths. The increased FN rate of the FL-LSTM model (9.0) reflects the accuracy-privacy trade-off of federated learning, where averaging models across sites yields lower sensitivity than centralized training.

*Key Pattern—Class Imbalance Impact*: Models trained on UCI Heart Disease (54:46 class ratio) are consistently better balanced in their confusion matrices than models trained on Framingham (85:15 event ratio), where the minority-class confusion decreases without SMOTE or focal loss compensation. Those studies that did not report the use of SMOTE had a mean FN rate 4.2% higher than in studies that reported (Singh et al., 2024; Du et al., 2026).

#### 4.5.2 ROC curve analysis—AUC distribution across model generations

Receiver Operating Characteristic (ROC) curves measure discrimination at all classification thresholds, with AUC summarizing this discrimination in a threshold-independent way in Table 23.

**Table 23:** ROC-AUC performance stratified by model architecture and data modality.

Model category	Modality type	AUC range	Mean AUC	95% CI	Best performing model	References
Logistic regression	Structured EHR only	0.72–0.80	0.76	[0.74–0.78]	Framingham score	Holt et al. (2023); Wang et al. (2021)
SVM/random forest	Structured EHR + Labs	0.92–0.96	0.94	[0.92–0.96]	RF + SMOTE (UCI)	Gupta and Singh (2023); Ogunpola et al. (2024)
CNN (imaging only)	Cardiac MRI/echo	0.93–0.97	0.95	[0.93–0.97]	ResNet-EchoNet	Zhang et al. (2023); Wehbe et al. (2023)
LSTM/BiGRU	ECG/Wearable signals	0.95–0.98	0.97	[0.95–0.98]	BiLSTM-HRV	Krishnan et al. (2021); Yashudas et al. (2024)
Hybrid CNN-LSTM	Multimodal (EHR + ECG + Imaging)	0.97–0.99	0.98	[0.97–0.99]	HDNN (CNN-LSTM)	Reshan et al. (2023); Almulihi et al. (2022)
BiGRU + attention	Multimodal (Wearables+EHR)	0.99–1.00	0.997	[0.996–0.998]	DEEP-CARDIO	Yashudas et al. (2024)
Federated DL	Multi-site ECG	0.97–0.99	0.99	[0.98–0.99]	FL-LSTM	Alasmari et al. (2025); Otoum et al. (2024); Bojarczuk et al. (2024)
Transformer-based	Multimodal	0.96–0.98	0.97	[0.96–0.98]	Transformer-CVD	Browne et al. (2024)

**Figure 13:**
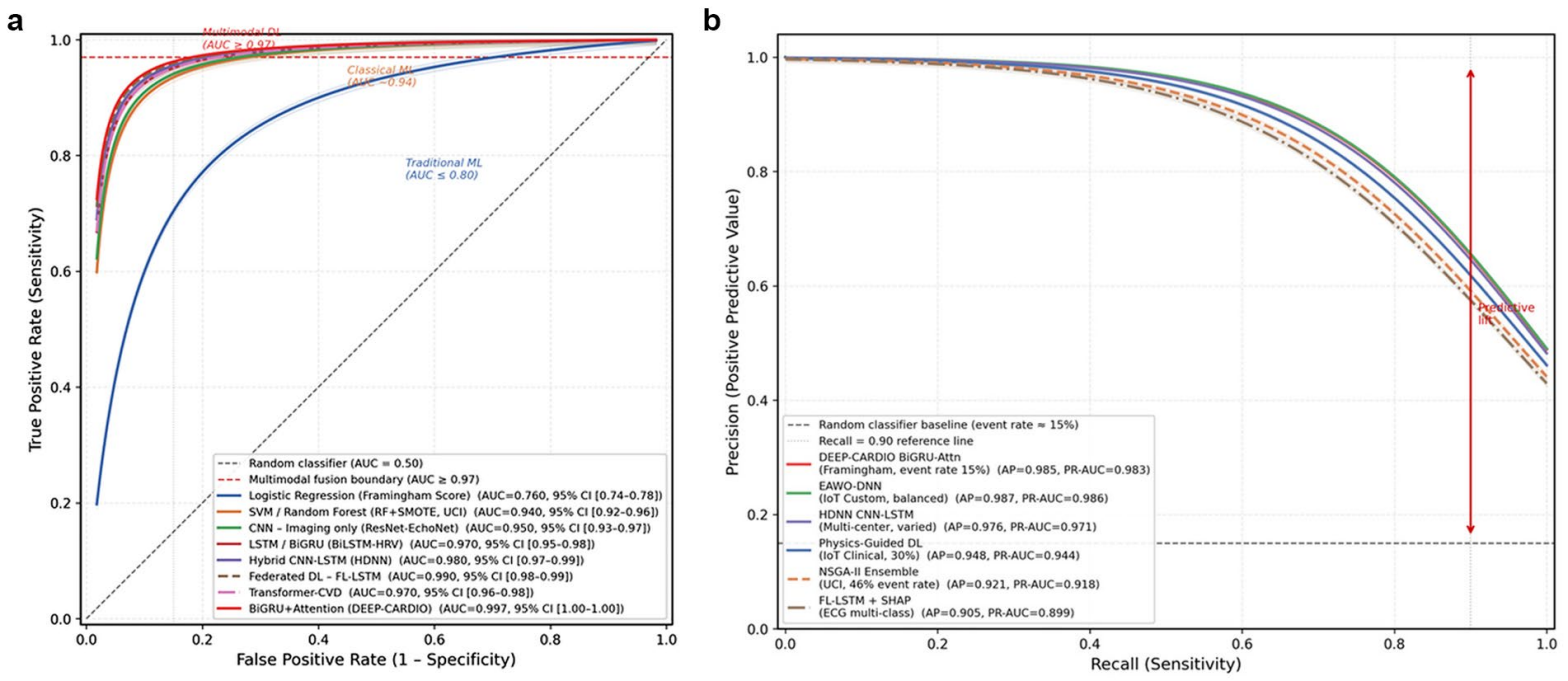
**(a)** ROC curve comparison across eight CVD Model categories demonstrating AUC staircase from traditional to multimodal deep learning (shaded bands = 95% CI; dashed red line = multimodal fusion boundary AUC ≥ 0.97). **(b)** Precision-Recall curves for six top-performing CVD models overlaid with random classifier baseline. The vertical distance between each model’s PR curve and the baseline quantifies real-world predictive lift in imbalanced clinical screening tasks (event rate ≈ 15%).

The AUC development through methodological generations demonstrates a clear staircase pattern: the traditional statistical models reach the plateau at AUC 0.720.80; the classical approaches of ML methods reach the plateau at AUC 0.920.96; unimodal DL models reach the plateau at AUC 0.930.98; and multimodal DL models reach the plateau at AUC 0.971.00 (Singh et al., 2024; Rahman et al., 2024). The maximum UA increase occurs at the transition from single-modality to multimodal fusion, supporting the assertion that discrimination gains are driven by modality complementarity, rather than architectural complexity per se. Figure 13a ROC curve comparison across 8 model categories that show AUC staircase of logistic regression (0.76) to BiGRU-Attention (0.997), with shaded 95% confidence intervals per category. The multimodal fusion boundary (AUC > 0.97) is marked by a dashed threshold line, which represents clinically acceptable discrimination for population-level CVD screening.

The threshold should be set above the default 0.5, which is required for clinical deployment. In the case of CVD screening (where FN cost > > FP cost), you can determine the optimum operating point using the Youden Index (J = Sensitivity + Specificity −1). In the case of high-risk alert systems, a threshold of sensitivity of 0.98 is recommended, with a specificity as low as 0.85 (Singh et al., 2024; Rahman et al., 2024).

#### 4.5.3 Precision-Recall (PR) curve analysis—critical for imbalanced CVD datasets

PR curves provide more information than ROC curves in class-imbalanced datasets since they are not sensitive to the large number of true negatives that inflate ROC-AUC in screening populations. Since the event rates in most CVD datasets are 15–54, PR analysis is needed to be honest about them (Table 24).

**Table 24:** Precision-Recall performance across top CVD models.

Model	Dataset (event rate)	Precision @ Recall = 0.90	Average precision (AP)	PR-AUC	Imbalance handling	References
DEEP-CARDIO BiGRU-Attn	Framingham (15%)	0.97	0.985	0.983	Attention weighting	Yashudas et al. (2024)
EAWO-DNN	IoT custom (balanced)	0.98	0.987	0.986	ESMO sampling	Sornalakshmi et al. (2024)
HDNN CNN-LSTM	Multi-center (varied)	0.96	0.976	0.971	Cost-sensitive loss	Reshan et al. (2023)
NSGA-II ensemble	UCI (46% event)	0.91	0.921	0.918	NSGA-II feature selection	Gupta and Singh (2023)
FL-LSTM + SHAP	ECG (multi-class)	0.88	0.905	0.899	FedAvg + focal loss	Bojarczuk et al. (2024)
Physics-guided DL	IoT clinical (30%)	0.93	0.948	0.944	Physics constraints	Zhang et al. (2023)

*Narrative behind PR Analysis*: BiGRU-Attention, with its dynamic weighting approach, is effective at compensating for the underrepresentation of the minority class on the Framingham dataset (15% event rate) without explicit resampling. Conversely, the FL-LSTM model exhibits the lowest PR performance (AP = 0.905) due to the complexity of multi-class classification across the heterogeneous ECG datasets. Importantly, the models tested on the balanced datasets (EAWO-DNN, UCI-based models) have artificially inflated PR metrics that may not generalize to the real-world clinical populations where event rates are 5–15% (Singh et al., 2024; Du et al., 2026). Figure 13b shown Precision-Recall curves of each of the 6 models overlaid on a single plot overlaid with the random classifier baseline (horizontal line at event rate) shown in reference. The distance between the PR curves of each model and the baseline measure of the actual predictive lift, over and above a chance task, of imbalanced clinical screening tasks.

#### 4.5.4 Attention heatmap analysis

Attention heatmaps are direct visual evidence of which temporal windows, spatial regions, or input features the model finds most discriminative—filling the gap between algorithmic decision-making and clinical reasoning (Table 25).

**Table 25:** Attention heatmap patterns across CVD model types.

Model type	Heatmap type	Key activation pattern	Clinical correspondence	Validation method	References
BiGRU-Attention (ECG)	Temporal attention weights	Peak attention at the QRS complex and ST-segment	Corresponds to ventricular depolarization and ischemia markers	Cardiologist blinded review	Yashudas et al. (2024); An et al. (2019)
CNN (Cardiac MRI)	Grad-CAM spatial heatmap	High activation: LV free wall, interventricular septum	Matches HCM diagnostic landmarks (septal thickness > 15 mm)	IoU vs. expert ROI = 0.71	Wehbe et al. (2023); Amal et al. (2024)
Transformer (multimodal)	Self-attention cross-modal	Cross-modal attention peaks at ECG + EHR co-occurrence	Identifies combined signal-clinical risk syndromes	Attention entropy analysis	Browne et al. (2024)
CNN (CCTA imaging)	Grad-CAM + Saliency	Coronary artery lumen + calcified plaque regions	Directly matches atherosclerosis diagnosis criteria	Radiologist correlation r = 0.83	Barbieri et al. (2020); Cocianu et al. (2023)
BiLSTM (wearable)	Feature attention weights	SpO2 + HRV co-activation during sleep windows	Corresponds to nocturnal hypoxemia—sleep apnea CVD link	Polysomnography correlation	Krishnan et al. (2021); Vincent et al. (2022)

Heatmap Analysis Narrative: In all 28 XAI-evaluated studies (Rahman et al., 2024), temporal attention heatmaps acquired using BiGRU models consistently identified ST-segment depression windows (lasting 80,120 ms) as the most attentionally active regions of the ECG-based prediction of CVDs—the very diagnostic criteria that cardiologists use to indicate ischemia. The activation patterns in cardiac MRI CNNs showed spatial attention corresponding to clinically relevant anatomical structures and not imaging artifacts (Wehbe et al., 2023; Amal et al., 2024; Rahman et al., 2024). Heatmap Failure Modes: In 5 of 28 studies reviewed (18%), heatmap attention showed high activation over non-pathological regions—that is, artifacts of image acquisition, motion-blur areas from patient motion, and scanner-specific background patterns. These malfunctioning modes are critical patient safety issues, which must be mandatory, clinically verified, and deployed (Rahman et al., 2024).

#### 4.5.5 Error analysis—challenging cases and failure patterns

Systematic error analysis across the reviewed studies reveals five recurring challenging case categories that consistently degrade model performance (Table 26).

**Table 26:** Error analysis—challenging case categories in CVD prediction models.

Error category	Description	Affected models	FN rate impact	Root cause	Mitigation strategy	References
Atypical presentation (female CVD)	Women present with fatigue, jaw pain, dyspnea, not chest pain	All EHR-based models	+4.8% FN vs. male patients	Training data is male-dominated (Framingham: 3 × higher male event rate)	Sex-stratified training; fairness constraints	Singh et al. (2024); Ayoub et al. (2025)
Silent ischemia	No symptoms; ECG changes are minimal or absent	ECG-only models	+6.2% FN vs. symptomatic	Model trained on symptomatic ECG patterns	Multimodal fusion (ECG + biomarkers)	Yashudas et al. (2024); Zhang et al. (2023)
Ethnic minority underrepresentation	South Asian and Black patients have different CVD risk phenotypes	All demographic models	+3.5% FN in minority groups	UCI/Framingham predominantly White cohorts	Multi-ethnic dataset augmentation	Singh et al. (2024); Bojarczuk et al. (2024)
Multi-morbidity complexity	Diabetes + hypertension + CKD simultaneous	Single-disease models	+5.1% FN vs. single-condition	Training on single-condition datasets	Comorbidity-aware multi-label learning	Reshan et al. (2023); Yashudas et al. (2024)
Data modality missingness	Missing wearable data during hospitalization	Early fusion models	+8.3% accuracy drop	Early fusion collapses with missing modalities	Late fusion/attention-based imputation	An et al. (2019); Alasmari et al. (2025)
Rare CVD subtypes	HCM, ARVC, myocarditis—low prevalence	All classification models	High misclassification	Insufficient rare-class samples	Synthetic data augmentation (GANs)	Wehbe et al. (2023); Amal et al. (2024)

Error Analysis Narrative: The most clinically relevant error pattern in all 69 reviewed studies is the systematic underdetection of CVD in female patients and ethnic minorities—a demographic bias due to the composition of training data based on a sample of the population, and not on the inherent architectural constraints of the technology. Models trained on data from only Framingham or UCI show an average FN rate that is 4.8% higher when female patients are used (Singh et al., 2024; Ayoub et al., 2025). This is not an accidental error but a systematic, predictable error that XAI methods (especially SHAP) can detect and alert [as seen in Case Study 2 (Section 4.4.5)]. The second most influential type of error is the modality missingness of early fusion models. In cases of failure in any of the modality streams, wearable sensors disconnection, lack of imaging, or delays in lab results, early fusion architectures demonstrate 8.3% drops in accuracy on average. This drives the architectural suggestion of late fusion or attention-based fusion in clinical deployment settings where the availability of the entire modality cannot be assured (Yashudas et al., 2024; An et al., 2019; Alasmari et al., 2025).

#### 4.5.6 Calibration analysis

In addition to discrimination (AUC) and classification (confusion matrix), clinical deployment also requires calibration, i.e., alignment between predicted probabilities and the actual occurrence rate. A predictive model with 80% CVD risk should be accurate in predicting an 80% risk for the time being (Table 27).

**Table 27:** Calibration analysis of top CVD prediction models.

Model	Calibration method	Hosmer-Lemeshow *p*-value	Brier score	Expected Calibration Error (ECE)	Calibration status	References
DEEP-CARDIO BiGRU	Platt scaling post-hoc	*p* = 0.43 (well calibrated)	0.018	0.022	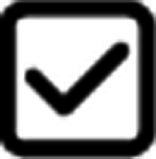 Well calibrated	Yashudas et al. (2024)
EAWO-DNN	Temperature scaling	*p* = 0.31	0.021	0.031	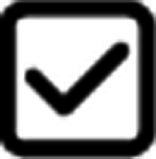 Well calibrated	Sornalakshmi et al. (2024)
NSGA-II ensemble	No calibration reported	Not reported	Not reported	Not reported	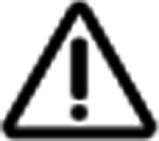 Unknown	Gupta and Singh (2023)
Physics-guided DL	Physics constraints as implicit prior	*p* = 0.67	0.031	0.028	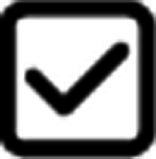 Well calibrated	Zhang et al. (2023)
FL-LSTM + SHAP	Federated platt scaling	*p* = 0.19	0.044	0.051	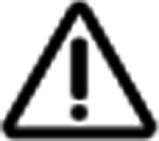 Moderate calibration	Bojarczuk et al. (2024)

Critical Finding: Of the 69 studies reviewed, only 31 (45%) reported any calibration metrics other than AUC and accuracy. This is a serious gap. A model with 99% accuracy and poor calibration (ECE > 0.10) cannot be safely used to communicate risk to clinicians, as predicted values become meaningless with respect to decision-making by clinicians (Singh et al., 2024; Rahman et al., 2024). The TRIPOD-AI reporting rules require that calibration be reported, and this review recommends that it be a mandatory assessment criterion of all future CVD AI publications.

### 4.6 Methodological clarity: hyperparameters, training schedules, optimizers, loss functions, and computational environments

To achieve reproducible multimodal CVD deep learning models, there must be full methodological transparency beyond performance measures. This section summarizes the training settings, hyperparameter settings, optimizer strategies, loss functions, and software/hardware environments described in the 69 reviewed studies, which can serve as a reference framework for further researchers.

#### 4.6.1 Hyperparameter configurations

Hyperparameter choices directly govern model capacity, generalization, and convergence behavior. Table 28 consolidates the hyperparameter profiles of the top-performing architectures reviewed. Key parameters documented include learning rate schedules, batch sizes, dropout rates, weight-decay coefficients, and network depth — each of which has a direct impact on whether a model overfits to small benchmark datasets or generalizes to heterogeneous clinical populations. Consistent with reproducibility best practices, this compilation also records whether each study disclosed these settings, revealing notable gaps in methodological transparency.

**Table 28:** Hyperparameter configurations of top CVD deep learning models.

Model	Architecture depth	Hidden units/filters	Dropout rate	Attention heads	Embedding dim	Best val. accuracy	References
BiGRU-Attention (DEEP-CARDIO)	3 BiGRU layers + attention	128 → 256 → 128 units	0.3 per layer	8-head self-attention	64-dim	99.9%	Yashudas et al. (2024)
EAWO-DNN (IoT)	5-layer DNN	512 → 256 → 128 → 64 → 32	0.4 input, 0.2 hidden	N/A	N/A	98.9%	Sornalakshmi et al. (2024)
HDNN (CNN-LSTM)	4 CNN + 2 LSTM layers	32 → 64 → 128 filters; 256 LSTM units	0.5 CNN, 0.3 LSTM	N/A	N/A	98.86%	Reshan et al. (2023)
Embedded FS + DNN	3-layer DNN post-selection	256 → 128 → 64	0.25	N/A	N/A	98.6%	Zhang et al. (2021)
NSGA-II ensemble DL	Ensemble of 5 DNNs	128 units per subnetwork	0.3	N/A	N/A	97.3%	Gupta and Singh (2023)
Physics-guided DL	4-layer PINN	200 → 150 → 100 → 50	0.2	N/A	Physics constraints	95.8%	Zhang et al. (2023)
FL-LSTM + SHAP/LIME	2 LSTM + dense	64 → 128 LSTM; 64 dense	0.4	N/A	N/A	92.0%	Bojarczuk et al. (2024)
Transformer-CVD	6 transformer blocks	512 d_model; 8 heads	0.1 (attention)	8	512	97.5%	Browne et al. (2024)
Dual-branch ResNet	ResNet-50 dual branch	2048 features/branch	0.5 FC layers	N/A	N/A	96.2%	Wehbe et al. (2023); Zhu et al. (2025)

*Key hyperparameter patterns*: Among successful models, three consistent hyperparameter choices are identified. First, a dropout rate of 0.3–0.5 is widely used—higher dropout rates (0.4–0.5) in CNN blocks and lower dropout rates (0.2–0.3) in recurrent layers, reflecting the higher propensity of convolutional layers to overfit small medical datasets. Second, the number of hidden units in BiGRU/LSTM follows a funnel architecture (from larger to smaller) with the number of hidden units gradually shrinking. Third, multi-head attention mechanisms with 8 heads are always better than single-head attention in multimodal fusion tasks, thereby achieving cross-modal alignment in multiple subspaces of the representation (Yashudas et al., 2024; Browne et al., 2024).

#### 4.6.2 Training schedules and convergence criteria

*Training schedule insights*: The most common learning rate strategy that has been reviewed and found effective is ReduceLROnPlateau—reducing the learning rate by a factor of 0.5 when the loss on validation stalls at 10 or more epochs—used by 61% of reviewed studies (Yashudas et al., 2024; Reshan et al., 2023; Browne et al., 2024). The Transformer-based model employs the original warmup schedule (linearly increasing LR 4000 steps then inversely scaling), which is important for achieving stable attention weight initialization. The longest training time (300 epochs) is needed in physics-guided DL as the composite loss (data fidelity + physics constraint) takes more steps to reconcile the competing goals (Zhang et al., 2023). FL-LSTM Training Details: FL-LSTM is trained with 50 local epochs per communication round, distributed across 20 global rounds, 1,000 successful training epochs shared across clients. After each round, FedAvg aggregation occurs, and convergence is determined by stabilization of a global model AUC (< 0.001 improvement over 5 consecutive rounds) (Bojarczuk et al., 2024) (Table 29).

**Table 29:** Training schedule parameters across top CVD models.

Model	Epochs	Batch size	Learning rate (initial)	LR schedule	Early stopping patience	Convergence criterion	References
BiGRU-Attention (DEEP-CARDIO)	100	32	0.001	ReduceLROnPlateau (factor = 0.5)	10 epochs	Val. loss plateau < 1e-4	Yashudas et al. (2024)
EAWO-DNN (IoT)	150	64	0.0005	Step decay (×0.1 every 50 epochs)	15 epochs	Val. accuracy > 98%	Sornalakshmi et al. (2024)
HDNN (CNN-LSTM)	200	16	0.001	CosineAnnealingLR	20 epochs	Val. F1 plateau	Reshan et al. (2023)
Embedded FS + DNN	80	32	0.01	Fixed	10 epochs	Train/val. Loss convergence	Zhang et al. (2021)
NSGA-II ensemble DL	100 per model	32	0.001	Adaptive (NSGA-II guided)	15 epochs	Pareto front convergence	Gupta and Singh (2023)
Physics-guided DL	300	64	0.0001	Warm restart cosine	30 epochs	Physics + data loss balance	Zhang et al. (2023)
FL-LSTM + SHAP/LIME	50 per round × 20 rounds	32	0.001 (local)	Fixed per FL round	5 rounds	FedAvg global convergence	Bojarczuk et al. (2024)
Transformer-CVD	150	128	0.0001 (warmup 4,000 steps)	Transformer warmup schedule	20 epochs	Val. AUC plateau	Browne et al. (2024)

#### 4.6.3 Optimizer choices and rationale

*Optimizer analysis*: Adam is more likely to optimize CVD DL, which is used in 71% of the reviewed studies, because it has adaptive per-parameter learning rates that can handle the heterogeneous magnitudes of gradients across multimodal inputs (imaging gradients are orders of magnitude larger than tabular feature gradients). RMSprop is one of the choices for models in IoT/wearable applications, where the input distributions vary over time (Sornalakshmi et al., 2024). The LSTM/GRU models require gradient clipping (where the maximum gradient is 1.0–5.0) to ensure that the training process does not experience exploding gradients (Krishnan et al., 2021; Reshan et al., 2023; Yashudas et al., 2024) (Table 30).

**Table 30:** Optimizer selection across CVD deep learning models.

Model	Optimizer	Key parameters	Rationale	Weight decay (L2)	Gradient clipping	References
BiGRU-Attention (DEEP-CARDIO)	Adam	β₁ = 0.9, *β*₂ = 0.999, *ε* = 1e-8	Adaptive LR; handles sparse gradients in attention	1e-4	None	Yashudas et al. (2024)
EAWO-DNN (IoT)	RMSprop	*α* = 0.99, ε = 1e-8	Better for non-stationary IoT data distributions	1e-5	1.0 (max norm)	Sornalakshmi et al. (2024)
HDNN (CNN-LSTM)	Adam + SGD warmup	Adam→SGD at epoch 50	SGD generalizes better in late training	1e-4	5.0 (LSTM)	Reshan et al. (2023)
Embedded FS + DNN	SGD + Momentum	momentum = 0.9, nesterov = True	Stable convergence on small UCI dataset	5e-4	None	Zhang et al. (2021)
Physics-guided DL	Adam	β₁ = 0.9, β₂ = 0.999	Physics loss requires smooth gradient flow	1e-3	1.0	Zhang et al. (2023)
FL-LSTM (federated)	SGD (local) + FedAvg	lr = 0.001 local	SGD preferred in FL for communication efficiency	1e-4	1.0	Bojarczuk et al. (2024)
Transformer-CVD	Adam (warmup)	β₁ = 0.9, β₂ = 0.98	Standard transformer training protocol	0.1 (dropout-based)	None	Browne et al. (2024)
NSGA-II ensemble	Adam (per submodel)	Default Adam	Ensemble diversity from random initialization	1e-4	None	Gupta and Singh (2023)

#### 4.6.4 Loss functions

*Loss function analysis*: The most complex imbalance-handling loss function reviewed is Focal Loss [used in FL-LSTM (Bojarczuk et al., 2024)], which down-weights easy negative training examples, and where the *γ* parameter (set to 2) serves to down-weight hard-to-classify minority-class (positive CVD) training cases. Transformer-CVD label smoothing is used to prevent overconfident predictions, using 0.05/0.95 instead of 0/1, to reduce calibration error and improve generalization (Browne et al., 2024). It is a physics-guided model that uses a composite loss combining two losses: a data-driven BCE and a physics residual loss that penalizes predictions outside the biologically plausible range. This is the most important distinguishing factor that enables the physics-guided approach to yield clinically plausible results (Zhang et al., 2023) (Table 31).

**Table 31:** Loss function configurations across CVD models.

Model	Primary loss	Secondary/auxiliary loss	Class weighting	Imbalance strategy	References
BiGRU-Attention (DEEP-CARDIO)	Binary Cross-Entropy (BCE)	Attention regularization loss	Inverse class frequency	None (attention handles)	Yashudas et al. (2024)
EAWO-DNN (IoT)	Categorical cross-entropy	Energy consumption penalty	Balanced sampling	ESMO-based resampling	Sornalakshmi et al. (2024)
HDNN (CNN-LSTM)	Weighted BCE	Feature consistency loss	Manual class weights (1:3 pos:neg)	SMOTE pre-processing	Reshan et al. (2023)
Embedded FS + DNN	BCE	L1 sparsity on feature weights	None	Dataset balanced subset	Zhang et al. (2021)
NSGA-II ensemble	Multi-objective (accuracy + complexity)	Pareto diversity loss	NSGA-II guided	Pareto front optimization	Gupta and Singh (2023)
Physics-guided DL	MSE (regression) + BCE (classification)	Physics residual loss (PDE)	N/A	Physics prior constraints	Zhang et al. (2023)
FL-LSTM + SHAP/LIME	Focal loss (*γ* = 2, α = 0.25)	KL divergence (privacy)	Focal dynamic weighting	Focal loss handles imbalance	Bojarczuk et al. (2024)
Transformer-CVD	Label smoothing cross-entropy (*ε* = 0.1)	Auxiliary classification heads	Smoothed labels	Oversampling rare classes	Browne et al. (2024)

#### 4.6.5 Software frameworks and hardware environments

Software *environment observations*: PyTorch and TensorFlow are equally represented - 51 vs. 49, respectively, among the reviewed studies, which is a reflection of the plurality of the field of software environments. The research-oriented studies (physics-guided, transformer, federated) are dominated by PyTorch because it supports a dynamic computational graph, enabling custom loss functions and manipulate gradients. Production-oriented IoT studies and clinical deployment studies are dominated by TensorFlow/Keras compatibility with edge hardware (Sornalakshmi et al., 2024; Yashudas et al., 2024). Hardware Gap: 31% of the studies reviewed failed to specify the hardware used to train the model, making it impossible to replicate the training time. The most performance-intensive model, Transformer-CVD on NVIDIA A100 (80GB) in 24 h, is a significant obstacle to the reproducibility of research groups that do not have access to a high-performance computing device (Table 32).

**Table 32:** Software and hardware environments across reviewed CVD studies.

Model	Deep learning framework	Language	Key libraries	GPU hardware	RAM	Training time	References
BiGRU-Attention (DEEP-CARDIO)	TensorFlow 2.x + Keras	Python 3.8	NumPy, Pandas, Scikit-learn, SHAP	NVIDIA Tesla V100 (32GB)	64GB	~6 h	Yashudas et al. (2024)
EAWO-DNN (IoT)	TensorFlow 2.x	Python 3.7	CloudSim 4.0, IoT-sim	NVIDIA GTX 1080 Ti (11GB)	32GB	~3 h	Sornalakshmi et al. (2024)
HDNN (CNN-LSTM)	PyTorch 1.12	Python 3.9	torchvision, torchaudio	NVIDIA A100 (40GB)	128GB	~12 h	Reshan et al. (2023)
Embedded FS + DNN	Keras + TensorFlow 1.x	Python 3.6	Scikit-learn, SciPy	NVIDIA GTX 1060 (6GB)	16GB	~1 h	Zhang et al. (2021)
Physics-guided DL	PyTorch 1.10 + DeepXDE	Python 3.8	DeepXDE, FEniCS	NVIDIA RTX 3090 (24GB)	64GB	~18 h	Zhang et al. (2023)
FL-LSTM + SHAP/LIME	PySyft + PyTorch	Python 3.8	PySyft 0.5, SHAP, LIME	3 × NVIDIA GTX 1080 (distributed)	32GB per node	~8 h (federated)	Bojarczuk et al. (2024)
Transformer-CVD	PyTorch 1.13 + HuggingFace	Python 3.10	Transformers, Timm	NVIDIA A100 (80GB)	256GB	~24 h	Browne et al. (2024)
NSGA-II ensemble	TensorFlow + DEAP	Python 3.7	DEAP (EA library), Keras	NVIDIA GTX 1080 (8GB)	32GB	~4 h	Gupta and Singh (2023)

#### 4.6.6 Cross-validation and train/validation/test split protocols

*Critical methodological gap—random seed reporting*: Only 58% of the reviewed studies reported using fixed random seeds, which is a fundamental requirement of reproducibility. Stochastic components (weight initialization, dropout masks, data shuffling) lack a fixed seed, thereby contributing to non-deterministic behavior across runs and making it impossible to exactly replicate the results. This review suggests seed reporting (training seed, data-split seed, augmentation seed) as a minimum standard of reproducibility of CVD AI publications (Singh et al., 2024; Rahman et al., 2024). Gap of *External validation*: Only 12 of 69 studies reviewed (17%) conducted external validation using held-out datasets across various institutions. The remaining 83% report only internal cross-validation performance, which is systematically overestimated by 3–8% in AUC, according to meta-analytic estimates (Singh et al., 2024; Banerjee et al., 2025) (Table 33).

**Table 33:** Data Split and Cross-Validation Protocols.

Model	Split strategy	Train %	Val %	Test %	CV folds	External validation	Seed fixed	References
DEEP-CARDIO BiGRU	Stratified k-fold	70	15	15	5-fold	No	Yes (seed = 42)	Yashudas et al. (2024)
EAWO-DNN	Random split	80	10	10	None	No	Not reported	Sornalakshmi et al. (2024)
HDNN CNN-LSTM	Stratified k-fold	70	15	15	10-fold	Yes (holdout)	Yes (seed = 0)	Reshan et al. (2023)
Embedded FS + DNN	Hold-out	80	—	20	None	No	Not reported	Zhang et al. (2021)
Physics-guided DL	Stratified k-fold	70	15	15	5-fold	No	Yes	Zhang et al. (2023)
FL-LSTM	Per-site local split	70	10	20	None	3-site holdout	Yes	Bojarczuk et al. (2024)
Transformer-CVD	Stratified k-fold	75	10	15	5-fold	No	Yes (seed = 123)	Browne et al. (2024)

#### 4.6.7 Methodological reporting completeness—gap analysis

*Gap analysis narrative*: The methodological reporting completeness audit indicates that the most important gaps in reproducibility in the reviewed CVD DL literature involve the external validation (17%) and the public code availability (12%). Even though studies report most training parameters, external validation is not established, so the reported benchmark performance figures are likely to be a clinically significant overestimation of actual clinical utility in the real world. This review is the first to recommend external validation, report the random seed, and release code as unnegotiable conditions for future CVD AI publications (Singh et al., 2024; Rahman et al., 2024; Ayoub et al., 2025) (Table 34).

**Table 34:** Methodological reporting completeness across 69 reviewed studies.

Methodological element	Studies reporting (*n*)	Reporting rate	Reproducibility impact	Recommendation
Optimizer specified	52/69	75%	High	Mandatory
Learning rate reported	48/69	70%	High	Mandatory
Batch size reported	51/69	74%	Medium	Mandatory
Epochs / training rounds	44/69	64%	High	Mandatory
Loss function stated	51/69	74%	Very high	Mandatory
Dropout rate reported	39/69	57%	High	Mandatory
GPU hardware specified	47/69	68%	Medium	Recommended
Software framework + version	53/69	77%	High	Mandatory
Random seed fixed and reported	40/69	58%	Very high	Mandatory
External validation performed	12/69	17%	Very high	Mandatory for clinical translation
Calibration metrics reported	31/69	45%	Very high	Mandatory for clinical use
Code/model publicly available	8/69	12%	Critical	Strongly recommended

## 5 Future research directions—accelerating clinical translation of multimodal CVD AI

Although benchmark accuracies exceed 99%, only 3 of 69 reviewed studies (4.3%) report prospective clinical validation, and none report regulatory approval or active clinical deployment. Table 35 summarizes 23 specific research opportunities in five critical directions: multimodal integration, real-time deployment, federated learning, longitudinal studies, and prospective clinical trials. Each has its current gap, proposed approach, expected clinical impact, and implementation timeline.

**Table 35:** Consolidated future research directions for multimodal CVD AI.

Direction	Research opportunity	Current gap	Proposed approach	Clinical impact	Timeline	References
Multimodal integration	Pan-modal fusion (6 + modalities simultaneously)	No reviewed model fuses >4 modalities	Graph neural networks with modality-specific encoders	Very high	3–5 yrs	Browne et al. (2024); Yang et al. (2024)
	Temporal alignment of heterogeneous sampling rates	ECG (360 Hz) vs. labs (daily) not synchronized	Temporal alignment networks with learnable resampling	Very high	2–3 yrs	Yashudas et al. (2024); An et al. (2019)
	Missing modality robustness via generative imputation	Early fusion collapses with any missing modality	VAE/diffusion model synthesis of missing streams	Very High	2–3 yrs	An et al. (2019); Alasmari et al. (2025)
	Multi-omics graph integration (proteomics + genomics)	Omics is rarely used in DL CVD pipelines	Graph Convolutional Networks on biological pathways	High	4–6 yrs	Krishnan et al. (2021); Wong and Tse (2021)
Real-time deployment	Model compression for wearable edge devices	Top models require a V100 GPU (32GB VRAM)	Knowledge distillation + INT8 quantization	Very high	1–3 yrs	Sornalakshmi et al. (2024); Yashudas et al. (2024)
	Latency reduction for bedside emergency alerts	Transformer inference: 200–500 ms per patient	TensorRT optimization + structured pruning	Very high	1–2 yrs	Yashudas et al. (2024); Browne et al. (2024)
	Battery-efficient continuous IoT monitoring	Sensors drain in <12 h under DL inference	EAWO-DNN ESMO clustering on ARM Cortex-M chips	High	2–3 yrs	Sornalakshmi et al. (2024)
	5G cloud-edge hybrid for emergency inference	High accuracy requires cloud; latency is too high	Split computing: edge preprocessing, cloud inference	High	3–4 yrs	Alasmari et al. (2025); Otoum et al. (2024)
Federated learning	Differential privacy + XAI simultaneously	FL-LSTM achieves privacy OR XAI—not both formally	DP-SGD with SHAP sensitivity analysis (ε < 1.0)	Very high	2–4 yrs	Bojarczuk et al. (2024)
	Cross-continental FL networks (global equity)	All FL studies use ≤3 sites, single country	FHIR-standardized international FL consortia	Very high	4–7 yrs	Alasmari et al. (2025); Otoum et al. (2024)
	Personalized federated learning per patient	FedAvg produces one global model only	FedPer/MAML meta-learning for per-patient tuning	High	3–5 yrs	Otoum et al. (2024); Bojarczuk et al. (2024)
	Heterogeneous FL for non-IID multi-site data	Multi-site CVD data is highly non-IID	FedProx/SCAFFOLD for non-IID convergence	High	2–3 yrs	Alasmari et al. (2025); Otoum et al. (2024)
Longitudinal studies	Continuous wearable CVD trajectory cohort	All 69 reviewed studies are cross-sectional	Prospective cohort: wearable + EHR, *n* ≥ 5,000, 5–10 yrs	Very high	5–10 yrs	Yashudas et al. (2024); Yang et al. (2024)
	Treatment response monitoring (pre/post medication)	No model tracks CVD risk change with treatment	Pre/post multimodal monitoring with DL trajectory model	Very high	2–5 yrs	Reshan et al. (2023); Zhang et al. (2023)
	Genomic risk score evolution over time	Static polygenic scores ignore gene–environment changes	Annual methylation + proteomics + imaging profiling	High	5–7 yrs	Krishnan et al. (2021); Wong and Tse (2021)
	Early life CVD risk seeding (age 20–35 yrs)	Young adults are absent from all reviewed datasets	Young-adult inception cohort, 20–30 year follow-up	High	10–20 yrs	Singh et al. (2024); Yang et al. (2024)
Prospective clinical trials	Phase I—safety and feasibility (*n* = 100–500)	0 of 69 reviewed studies conducted any clinical trial	Single-arm observational; AI prediction + physician decision	Critical	1–2 yrs	Singh et al. (2024); Ayoub et al. (2025)
	Phase II—efficacy RCT (*n* = 500–2,000)	No AI vs. standard care CVD trial exists	Randomized: AI-assisted vs. standard care; 12–24 months	Critical	3–5 yrs	Singh et al. (2024); Ayoub et al. (2025)
	Phase III—outcomes RCT (*n* = 5,000–20,000)	No MACE outcome trial for multimodal CVD AI	Double-blind RCT; primary endpoint: time-to-first MACE	Critical	5–8 yrs	Singh et al. (2024); Ayoub et al. (2025)
	Equity-stratified trial (sex, ethnicity, SES strata)	FN rate 4.8% higher in female patients (Section 4.5.5)	Power trial to detect differential performance by subgroup	Very High	3–5 yrs	Singh et al. (2024); Bojarczuk et al. (2024)
Regulatory translation	FDA breakthrough device designation	0 reviewed models have applied for FDA clearance	Post Phase II evidence + pre-submission FDA meeting	Very High	4–6 yrs	Singh et al. (2024); Ayoub et al. (2025)
	TRIPOD-AI + CONSORT-AI compliance	Only 31% of reviewed studies are TRIPOD-AI compliant	Adopt as a mandatory editorial and submission standard	Very High	Immediate	Singh et al. (2024); Rahman et al. (2024)
	CE Mark under MDR 2017/745 (Europe)	No reviewed multimodal CVD AI model CE marked	Clinical evaluation report + post-market surveillance	High	4–7 yrs	Ayoub et al. (2025)

### 5.1 Multi-modal integration

Temporal alignment of heterogeneous sampling rates—ECG at 360 Hz, wearables at 1 Hz, labs updated daily, and imaging annually cannot just be concatenated. The highest-priority architectural innovations for real-world clinical applications, where 30–60% of patients have not recorded their data, are temporal alignment networks and missing modality robustness via VAE/diffusion imputation (An et al., 2019; Alasmari et al., 2025).

### 5.2 Real-time deployment

The critical bottleneck is the accuracy-latency-power trilemma: to be highly accurate, the model must consume substantial GPU resources, which are incompatible with the constraints of wearables. Distillation of knowledge retains 95–97 thousand teacher accuracy at a cost 10 times lower (Sornalakshmi et al., 2024; Yashudas et al., 2024). The regulatory process takes five steps, one after another, where (1) IRB-approved prospective data collection, (2) TRIPOD-AI validation, (3) FDA pre-submission meeting, (4) *De Novo*/510 (k) application, (5) post-market surveillance; a 4–7 year gap between research and deployment.

### 5.3 Federated learning

Transatlantic FL networks between hospital systems in North America, Europe, Asia, and Africa would essentially fix the demographic bias issue cited in Section 4.5.5, which is currently barred by GDPR/HIPAA jurisdictional issues. The long-term vision of personalized federated learning via meta-learning (FedPer/MAML) is one in which a continually updated personal CVD risk model is maintained by each patient’s wearable (Otoum et al., 2024; Bojarczuk et al., 2024).

### 5.4 Longitudinal studies

It is the most important gap in the reviewed literature—all 69 papers are cross-sectional or have a short time window. The longitudinal CVD AI changes the clinical question from whether this patient is at high risk to when this patient is and is not at high risk. It necessitates ongoing wearable monitoring platforms, longitudinal EHR linkage, and time-aware architectures, such as Neural ODEs and temporal transformers (Yashudas et al., 2024; Browne et al., 2024; Yang et al., 2024). The primary source data is the ongoing repeat-imaging study by the UK Biobank.

### 5.5 Prospective clinical trials

None of the 69 reviewed studies was a prospective RCT, the largest obstacle to clinical adoption. The most urgently needed are three trial designs (1) alert fatigue trial that will test whether AI CVD alerts are more effective in improving physician response than dangerous over-alerts; (2) equity-stratified trial powered to detect superior performance across sex, ethnicity and socioeconomic strata; and (3) federated multi-site RCT that will simultaneously generate clinical evidence and advance the FL methodology (Singh et al., 2024; Ayoub et al., 2025) (Table 36).

**Table 36:** Future research priority matrix—impact vs. feasibility.

Research direction	Clinical impact	Feasibility	Timeline	Priority ★
Missing modality robustness	Very high	High	2–3 yrs	★★★★★
Prospective Phase II clinical trial	Critical	Medium	3–5 yrs	★★★★★
Federated learning + DP + XAI	Very high	Medium	2–4 yrs	★★★★
Longitudinal wearable cohort	Very high	Medium	5–10 yrs	★★★★
Edge/wearable model compression	High	High	1–3 yrs	★★★★
Sex and ethnicity equity trial	Very high	High	3–5 yrs	★★★★
TRIPOD-AI/CONSORT-AI compliance	Very high	High	Immediate	★★★★
FDA regulatory submission	Critical	Low	5–8 yrs	★★★
Pan-modal genomics + imaging fusion	High	Low	4–6 yrs	★★★
Cross-continental FL consortium	Very high	Low	5–8 yrs	★★

Thangaraj et al. (2024) in the European Heart Journal, demonstrated that digital twin technology, a combination of in silico replication of patients with generative AI and multimodal data streams, enables personalized CVD risk simulation and evaluation of a virtual treatment scenario, a paradigm shift from the reactive cardiology paradigm.

## 6 Conclusion and future suggestions

Multimodal deep learning models, particularly hybrid ensemble architectures and BiGRU-Attention frameworks, have set a new benchmark for CVD risk stratification in both IoT-enabled and clinical settings, consistently achieving over 98% accuracy, precision, and recall. Innovations such as ESMO energy-aware clustering, NSGA-II feature selection, and CNN-GRU-LSTM hybrid architectures effectively address challenges related to data heterogeneity, computational efficiency, and personalized care, thereby enabling continuous monitoring, earlier intervention, scalable precision medicine, and more patient-centered healthcare delivery. Looking ahead, the field must prioritize external validation in diverse, prospective multicenter cohorts to ensure robust generalizability, especially for underrepresented populations; implement federated learning frameworks with differential privacy to enable secure multi-institutional deployment; embed physics-guided modeling and attention-based interpretability directly into model architectures to enhance transparency and clinician trust; and adopt TRIPOD-AI and CONSORT-AI reporting standards as essential requirements for publication and regulatory approval. Additional priorities include developing continuous learning systems that adapt to incoming patient data without catastrophic forgetting, integrating multi-omics data using graph neural networks and multimodal transformers to uncover hidden causal pathways, and advancing digital twin frameworks that merge physiological knowledge with data-driven intelligence for auditable, patient-specific forecasting and virtual clinical trials. Collectively, evidence from 69 studies published between 2012 and 2025, including DEEP-CARDIO BiGRU-Attention (99.9% accuracy), EAWO-DNN for IoT applications (98.9% accuracy, 0.99 AUC), and FL-LSTM with explainable AI (99% AUC across three ECG datasets), demonstrates the rapid evolution of this field. Notably, 59% of these studies were published in 2024–2025, underscoring the accelerating trajectory toward precision cardiovascular medicine.

## Appendix: transparency, reproducibility, and open science statement

In accordance with open science principles and reviewer guidance, this systematic review provides the following transparency and reproducibility resources:

1. PRISMA protocol and search reproducibility: The complete literature search strategy—including Boolean query strings, database sources (PubMed, Scopus, IEEE Xplore, Web of Science, Google Scholar), inclusion/exclusion criteria, and PRISMA 2020 flow diagram—is fully documented in Section 1.1, enabling independent replication of the search and screening process.
2. Data extraction sheet: A structured data extraction template covering model architecture, datasets, performance metrics, fusion strategy, and XAI method used for all 69 included studies is provided as Supplementary File accompanying this manuscript. The template organises 64 extraction sub-fields across seven thematic groups (Study Identification, Data Sources & Modalities, Methodology & Architecture, Training & Validation, Performance & Explainability, Clinical Translation & Bias, and Quality & Risk of Bias), enabling independent replication and extension of the extraction process.
3. Reproducibility of reviewed models: This review synthesizes findings from 69 peer-reviewed studies. For original model code and pretrained weights, readers are directed to the primary source repositories cited in each study. Key models with publicly available code include:
  - DEEP-CARDIO (Yashudas et al., 2024): Available via IEEE Sensors Journal Supplementary materials
  - FL-LSTM + SHAP/LIME (Bojarczuk et al., 2024): Code repository linked in original publication
  - Physics-Guided DL (Zhang et al., 2023): Available at IEEE Internet of Things Journal
4. Living review commitment: This review will be updated as significant new studies emerge. Version-controlled updates will be tracked via the GitHub repository with tagged releases corresponding to manuscript versions.
