# Supplementary material for: Deep learning for cardiovascular disease: a comprehensive review of detection and risk forecasting
Source: Front Artif Intell. 2026 Jun 26;9:1840804. doi: 10.3389/frai.2026.1840804 (PMC13350234; doi:10.3389/frai.2026.1840804)
Supplement: Supplementary file 1 [file Data_Sheet_1.pdf]

Supplementary File — Data Extraction Sheet

Deep learning for cardiovascular disease: a comprehensive review of detection and risk forecasting

Frontiers in Artificial Intelligence | Systematic Review | 69 Studies | 2012–2026

1. Overview

All 69 studies included in this systematic review are recorded on this completed data extraction sheet, which contains key bibliographic, methodological and performance data. Data are directly taken from the reference list, Tables 9, 11, 12, 14, 17 and associated performance analyses from the manuscript. Data reported as NR are not included in the paper reviewed. Studies with green shading had ≥99% accuracy or ≥ 0.99 AUC. Quality scores (Q, 0–10) are based on the size of the dataset, external validation, transparency of XAI, reproducibility, and evidence of clinical translation.
Studies are categorised under groups of categories (A to G) to help navigation. Review/survey articles were included as they influenced the synthesis methodology. The 69 included studies span 2012–2026, with 59% (n=41) published in 2024–2025.

2. Complete Data Extraction Table (All 69 Studies)

Legend: Green rows = top-performing models (Acc ≥99% or AUC ≥0.99). NR = Not Reported. Blue text in Architecture column = primary model name. Sub-headers (dark blue rows) = thematic category groups A–G. Q = Reviewer quality score (0–10).

S.No	Ref	First Author (Year)	Year	Journal / Venue	Dataset(s)	Data Modalities	Architecture / Model	Fusion Level	Acc (%)	AUC	F1 (%)	XAI Method	Key Notes / Limitations	Q
A — IoT & Wearable-Centric Models (Studies 1–8)														
1	[1]	Sornalakshmi et al.	2024	J. Biomolecular Structure & Dynamics	CloudSim + Real IoT	IoT wearable + EHR	EAWO-DNN (Optimized DNN)	Late	98.9	>0.99	98.8	None	IoT edge deployment; energy-aware optimization; no external validation	7
2	[2]	Gupta & Singh	2023	Expert Systems	UCI Heart Disease	EHR/Clinical	EDL-NSGA-II (Ensemble DL + NSGA-II FS)	Late	97.3	0.97	92.1	None	Feature selection via NSGA-II; UCI only; no imaging	6
3	[3]	Zhang et al.	2021	J. Healthcare Engineering	Kaggle + UCI	EHR/Clinical	Embedded FS + DNN	Early	98.6	0.983	98.3	None	Embedded feature selection; single modality limitation	6
4	[4]	Krishnan et al.	2021	Int. J. Electrical & Computer Eng.	Echo + HRV datasets	ECG/Physiological + Imaging	CNN + BiLSTM hybrid	Intermediate	97.5	NR	NR	None	Hybrid RNN-GRU; limited dataset diversity	6
5	[5]	Reshan et al.	2023	IEEE Access, vol.11	Large Multi-center	EHR + Clinical	HDNN (CNN-LSTM)	Intermediate	98.9	0.91	98.7	None	Robust hybrid DNN; multi-center data; no XAI	7
6	[6]	Almulihi et al.	2022	Diagnostics, vol.12	UCI + MIMIC	EHR/Clinical	Ensemble DL (Hybrid)	Late	97.5	NR	NR	None	Early detection	6

												focus; ensemble approach; limited modalities	
7	[7]	Nancy et al.	2022	Electronics, vol.11	IoT-Cloud platform	IoT wearable + EHR	IoT-DL / LSTM-CNN	Intermediate	87.6	NR	NR	None	IoT-cloud integration; lower accuracy vs multimodal peers	6
8	[8]	Yashudas et al.	2024	IEEE Sensors Journal, vol.24	Framingham + Statlog	Wearable + EHR	DEEP-CARDIO: BiGRU + Attention	Attention	99.9	0.997	98.7	Attention Viz.	Best-in-review model; IoT wearable fusion; attention XAI; no prospective trial	9
B — Traditional Statistical & Single-Modality Baselines (Studies 9–18)														
9	[9]	Holt et al.	2023	Eur. J. Preventive Cardiology	76,000-patient cohort	Clinical/EHR	Statistical risk equations	Traditional	NR	0.82	NR	None	Validation study; traditional statistical baseline; large cohort	7
10	[10]	Krittanawong et al.	2019	European Heart Journal	Multiple clinical datasets	EHR + Imaging	Deep learning review (multiple)	Review	NR	NR	NR	None	Primer/review; no original model; foundational reference	5
11	[11]	Hathaway et al.	2021	Computers in Biology & Medicine, vol.139	MESA cohort	EHR + Clinical	Deep Neural Survival Networks	Late	NR	0.84	NR	None	Survival analysis focus; MESA dataset; limited modalities	7
12	[12]	Stahlschmidt et al.	2022	Briefings in Bioinformatics, vol.23	Multiple biomedical	Multi-omics + EHR + Imaging	Multimodal DL review	Review	NR	NR	NR	None	Systematic review; no original model; methodology reference	8
13	[13]	Gao et al.	2020	Neural Computation, vol.32	Multiple benchmark	Multi-source	Survey (m-RNN, DBN, CNN)	Review	NR	NR	NR	None	Survey of multimodal DL fusion; foundational architecture reference	8

14	[14]	Zhang et al.	2023	IEEE Internet of Things Journal, vol.10	IoT + Clinical	IoT + Clinical/Physiological	Physics-Guided DL	Intermediate	>95	0.96	95.3	Physics-guided	Physics-guided constraints; IoT deployment; hemodynamic constraints in loss	8
15	[15]	An et al.	2019	IEEE/ACM Trans. Computational Biology, vol.18	Multiple EHR datasets	EHR + Clinical	Attention-based DNN	Attention	NR	0.93	NR	Attention Viz.	High-risk prediction; attention mechanisms; earlier architecture	7
16	[16]	Fathima & Fasla	2024	Computer Methods in Biomechanics & Biomed. Eng.	Multiple public datasets	EHR + Clinical	Review of AI algorithms	Review	NR	NR	NR	None	Comprehensive review; no original model	5
17	[17]	Wong & Tse	2021	Frontiers in Cardiovascular Medicine, vol.8	Clinical biomarker datasets	EHR + Biomarkers	Biomarker prediction models	Traditional	NR	NR	NR	None	Biomarker focus; limited deep learning	6
18	[18]	Yun et al.	2022	Reviews in Cardiovascular Medicine, vol.23	Genomic datasets	Genomics/Omics	Genetic risk score models	Traditional	NR	NR	NR	None	Systematic review of genetic risk scores; no DL model	6
C — IoMT, Imaging & Specialist Modalities (Studies 19–29)														
19	[19]	Mulani et al.	2025	J. Pharmacology & Pharmacotherapeutics	IoMT platform	IoT + EHR	ML-IoMT structure (DL-based)	Early	NR	NR	NR	None	IoMT focus; novel 2025 publication; limited performance details	6
20	[20]	Wang et al.	2017	IEEE Trans. Medical Imaging, vol.36	Mammogram datasets (BAC)	Medical Imaging (Mammography)	CNN for BAC detection	Early	NR	NR	NR	None	Mammogram CVD screening; early seminal work; single modality imaging	7
21	[21]	Haq et al.	2021	Cardiovascular Diagnosis & Therapy, vol.11	Clinical imaging datasets	EHR + Imaging	AI-based CVD prediction models	Review	NR	NR	NR	None	Personalized CVD medicine review; imaging AI focus	6
22	[22]	Bhagawati et al.	2023	Cardiovascular Diagnosis & Therapy, vol.13	Multiple clinical datasets	EHR + Imaging + Biomarkers	DL review for CVD risk stratification	Review	NR	NR	NR	None	Comprehensive risk stratification review;	7

													multimodal coverage	
23	[23]	Li et al.	2022	Biomedicines, vol.10	Multiple biomechanics datasets	EHR + Imaging + Biomechanics	AI + biomechanics hybrid	Intermediate	NR	NR	NR	None	Interplay of AI and biomechanics; novel modality integration	6
24	[24]	Sumalatha et al.	2024	IEEE Access, vol.12	Multiple ECG datasets	ECG/Physiological	DL for ECG analysis (review)	Review	NR	NR	NR	None	ECG-focused DL review; disease detection focus	6
25	[25]	Oh & Shim	2024	J. Clinical Oncology	Breast cancer survivor cohort	EHR + Clinical	CVD risk prediction DL model	Late	NR	NR	NR	None	Long-term breast cancer survivor CVD risk; novel population focus	7
26	[26]	Wehbe et al.	2023	JAMA Cardiology	Multiple imaging datasets	Medical Imaging	DL for cardiovascular imaging (review)	Review	NR	NR	NR	None	JAMA review; imaging AI comprehensive coverage	8
27	[27]	Barbieri et al.	2020	Int. J. Epidemiology, vol.51	National administrative DB	EHR + Administrative data	Survival analysis + DL combined	Late	NR	0.79	NR	None	Admin database CVD risk; survival analysis hybrid; no imaging	7
28	[28]	Cocianu et al.	2023	Electronics	Multiple benchmark	EHR/Clinical	Classical + Evolutionary + DL comparison	Comparison	NR	NR	NR	None	Comparative study; evolutionary algorithms included	6
29	[29]	Selvarathi & Varadhaganapathy	2023	Information Technology & Control, vol.52	Type 2 DM patient dataset	EHR + Clinical	DL CVD risk factor prediction	Early	NR	NR	NR	None	Diabetes comorbidity focus; specific population	6
D — Population, Behavioral & Comparative Studies (Studies 30–43)														
30	[30]	Sekar et al.	2012	IEEE Sensors Journal, vol.12	Multi-center cardiac datasets	EHR + Physiological	Fused Hierarchical Neural Networks	Intermediate	NR	NR	NR	None	Early seminal fused NN work; 2012 baseline architecture	6
31	[31]	Wang et al.	2021	Frontiers in Cardiovascular Medicine, vol.8	Preeclampsia patient cohort	EHR + Clinical + Biomarkers	ML-based CVD risk model	Late	NR	NR	NR	None	Women with preeclampsia specific; sex-	7

													stratified analysis	
32	[32]	Ordikhani et al.	2022	PLoS ONE, vol.17	Large Iranian population cohort	EHR + Clinical	Evolutionary ML algorithm	Late	NR	NR	NR	None	Evolutionary algorithm; population-specific; Iran cohort	6
33	[33]	Ahmad et al.	2021	Computers, Materials & Continua	Multiple benchmark	EHR + Clinical	Data+ML fusion architecture	Intermediate	NR	NR	NR	None	Fusion architecture study; benchmark datasets; no imaging	6
34	[34]	Amal et al.	2024	Frontiers in Radiology	Multiple imaging + EHR	EHR + Medical Imaging	AI multimodal imaging fusion	Late	NR	NR	NR	None	Imaging + EHR fusion; CVD care improvement focus	7
35	[35]	Sadr et al.	2024	Frontiers in Cardiovascular Medicine	Multiple clinical datasets	EHR + Clinical + Imaging	DL + ML holistic approach	Late	NR	NR	NR	None	Holistic DL+ML hybrid; broad modality coverage	7
36	[36]	Honi et al.	2024	Informatics in Medicine Unlocked	Multiple ECG datasets	ECG/Physiological	1D-CNN for CVD prediction	Early	NR	NR	NR	None	1D-CNN ECG focus; single modality; real-time potential	6
37	[37]	Amal et al.	2024	Frontiers in Radiology	Multiple imaging + EHR	EHR + Medical Imaging	AI multimodal imaging data fusion	Late	NR	NR	NR	None	Near-duplicate of [34]; imaging + EHR fusion	7
38	[38]	Addissouky et al.	2024	Probiologists	Multiple clinical datasets	EHR + Clinical	AI diagnostic approaches review	Review	NR	NR	NR	None	Recent AI review; broad CVD coverage; review article	5
39	[39]	Ayoub et al.	2025	JACC: Advances	Cancer patient cohort (ICI therapy)	EHR + Imaging + Biomarkers	Multimodal AI fusion model	Late	NR	NR	NR	None	Myocarditis prediction in cancer patients; novel 2025 ICI context	8
40	[40]	Khera et al.	2024	J. American College of Cardiology	Multiple clinical datasets	EHR + Imaging + Clinical	AI-based CVD care transformation	Review	NR	NR	NR	None	JACC perspective; clinical AI translation focus	8

41	[41]	Wang et al.	2024	Frontiers in Psychiatry	Behavioral pattern datasets	EHR + Behavioral	DL behavior-based CVD prediction	Early	NR	NR	NR	None	Behavioral data focus; novel modality; psychiatric comorbidity	6
42	[42]	Dharmarathne et al.	2024	Patterns	Multiple clinical datasets	EHR + Clinical	Explainable ML + user-centric design	Late	NR	NR	SHAP	Human-centered XAI design; explainability priority; user trust focus	7	NR
43	[43]	Ogunpola et al.	2024	Diagnostics, vol.14	Multiple benchmark	EHR/Clinical	ML predictive models comparison	Comparison	NR	NR	NR	None	ML comparison study; heart disease detection; no deep fusion	6
E — Multimodal Fusion Architecture Studies (Studies 44–54)														
44	[44]	Zhu et al.	2023	Proc. ACM Int. Conference	Multi-modal benchmark	EHR + ECG + Imaging	Multi-Branch Residual Networks	Intermediate	NR	NR	NR	None	Multi-branch residual architecture; multi-modal benchmark	7
45	[45]	Singh et al.	2024	J. Cardiovascular Computed Tomography	Multiple clinical + imaging	EHR + Imaging	AI CVD risk prediction review	Review	NR	NR	NR	None	CT-focused review; future directions; imaging AI focus	7
46	[46]	Alasmari et al.	2025	Frontiers in Cardiovascular Medicine	Multi-site federated data	EHR + ECG + Imaging	Federated learning multimodal	Late/Federated	96.7	0.97	96.1	None	Federated early cardiac detection; privacy-preserving; 2025	8
47	[47]	Evangelin Sonia et al.	2023	Automation & Remote Control	Multiple clinical	EHR + Clinical	Multi-modal integrated DNN	Intermediate	NR	NR	NR	None	Multi-modal DNN; Automation journal; limited detail available	6
48	[48]	Shaik et al.	2024	Information Fusion, vol.101	Multiple healthcare datasets	EHR + Imaging + Wearable	Multimodal information fusion survey	Review	NR	NR	NR	None	Smart healthcare fusion survey; broad modality review	7

49	[49]	Meder et al.	2025	Frontiers in Cardiovascular Medicine	Population health datasets	EHR + Imaging + Population	AI for CVD population health	Review	NR	NR	NR	None	Population health AI focus; state-of-the-art 2025 review	7
50	[50]	Zhu et al.	2025	Biomed. Signal Processing & Control, vol.99	Multi-modal benchmark	ECG + EHR + Clinical	DDR-Net (Dual-scale DL)	Intermediate	NR	NR	NR	None	Multi-modal DDR-Net; note: citation details flagged for verification	7
51	[51]	El-Sofany et al.	2024	Scientific Reports	Multiple clinical datasets	EHR + Clinical	Feature selection-based DL	Early	NR	NR	NR	None	Proposed technique with feature selection; Scientific Reports 2024	6
52	[52]	Li et al.	2024	Computers in Biology & Medicine, vol.174	Multiple medical datasets	EHR + Imaging + Signal	DL-based information fusion review	Review	NR	NR	NR	None	Medical multimodal classification review; comprehensive coverage	7
53	[53]	Jaltotage et al.	2024	Canadian Journal of Cardiology Open	Multiple clinical + imaging	EHR + Imaging + Clinical	AI multimodal CVD management	Review	NR	NR	NR	None	Canadian context; multimodal management review; clinical translation	7
54	[54]	Zhang et al.	2025	Alexandria Engineering Journal, vol.127	HF patient IoT datasets	EHR + Imaging + IoT	GNN + CNN multimodal fusion	Intermediate	NR	NR	NR	None	Heart failure IoT monitoring; GNN+CNN; authors corrected per proof	8
F — XAI, Explainability & Clinical Translation (Studies 55–68)														
55	[55]	Venkatesh et al.	2024	Heliyon, vol.10	Multiple health record datasets	EHR + Clinical + Imaging	Automatic diagnostic DL model	Late	NR	NR	NR	None	Multimodal EHR prognosis; automatic diagnosis; Heliyon 2024	7
56	[56]	Bojarczuk et al.	2024	Applied Soft Computing, vol.141	Multiple clinical datasets	EHR + Clinical	Hybrid explainable DL	Late	NR	NR	SHAP+LIME	Hybrid XAI DL; explainability priority; also cited as [67]	7	NR

57	[57]	Otoum et al.	2024	IEEE Internet of Things Journal	Multi-site IoT health data	IoT + EHR + ECG	Federated DL CVD screening	Late/Federated	NR	NR	NR	None	Federated privacy-preserving CVD; IoT smart healthcare	8
58	[58]	Krishna et al.	2023	IEEE Access	Multiple CVD datasets	EHR + ECG + Imaging	Ensemble DL multimodal	Late	NR	NR	NR	None	Ensemble DL multimodal detection; also cited as [68] duplicate	7
59	[59]	Saha et al.	2023	Artificial Intelligence Review	Multiple CVD datasets	EHR + Imaging + Clinical	XAI multimodal survey for CVD	Review	NR	NR	NR	Multiple XAI	Comprehensive XAI multimodal survey; risk stratification focus	8
60	[60]	Yang et al.	2024	Frontiers in Radiology	Longitudinal imaging datasets	Medical Imaging (longitudinal)	AI CVD risk modeling	Late	NR	NR	NR	None	Longitudinal multimodal imaging; AI risk modeling; also cited as [65]	7
61	[61]	Dehghani et al.	2025	IEEE Reviews in Biomedical Engineering	Multiple CVD datasets	EHR + ECG + Imaging	Explainable AI multimodal CVD	Late	NR	NR	NR	Grad-CAM+SHAP	XAI multimodal CVD 2025; IEEE Reviews; top-tier venue	8
62	[62]	Browne et al.	2024	Scientific Reports, vol.14	Multiple clinical datasets	EHR + Clinical	Transformer-based attention models	Attention	96-98	NR	NR	Attention Viz.	Citation flagged for verification; transformer CVD risk attention	7
63	[63]	Singh et al.	2024	J. Cardiovascular Computed Tomography	Multiple clinical + imaging	EHR + Imaging	AI CVD risk prediction review	Review	NR	NR	NR	None	Near-duplicate of [45]; future directions review	7
64	[64]	Rahman et al.	2024	BMC Medical Informatics & Decision Making	Multiple multimodal datasets	EHR + Imaging + Clinical	Multimodal data fusion precision cardiology	Late	NR	NR	NR	None	Precision cardiology fusion review; trends and prospects	7

65	[65]	Yang et al.	2024	Frontiers in Radiology	Longitudinal imaging datasets	Medical Imaging (longitudinal)	AI CVD risk modeling	Late	NR	NR	NR	None	Duplicate of [60]; longitudinal imaging AI risk	7
66	[66]	Du et al.	2026	Frontiers in Medicine, vol.12	Cardiac MRI datasets	Medical Imaging (cardiac MRI)	CASNet (curvature-aware MRI segmentation)	Intermediate	NR	NR	NR	None	Cardiac MRI segmentation; 2026 publication; attention-driven encoding	8
67	[67]	Bojarczuk et al.	2024	Applied Soft Computing, vol.141	Multiple clinical datasets	EHR + Clinical	Hybrid explainable DL + FL-LSTM	Late	92.0	0.99	91.0	SHAP+LIME	FL-LSTM with SHAP+LIME; 99% AUC on 3 ECG datasets; duplicate of [56]	8
68	[68]	Krishna et al.	2023	IEEE Access	Multiple CVD datasets	EHR + ECG + Imaging	Ensemble DL multimodal	Late	NR	NR	NR	None	Duplicate of [58]; ensemble multimodal detection	7
G — Digital Twin, Cross-Domain & Review Studies (Studies 69–78)														
69	[69]	Haq et al.	2026	Biomed. Signal Processing & Control, vol.119	Multiple cardiac imaging datasets	EHR + Imaging (multiple modalities)	DL advancements review for CVD imaging	Review	NR	NR	NR	None	2026 review; imaging modality challenges; future perspectives	7
70	[70]	Ayoub et al.	2025	JACC: Advances	Cancer ICI therapy cohort	EHR + Imaging + Biomarkers	Multimodal AI fusion model	Late	NR	NR	NR	None	Myocarditis + adverse events prediction; near-duplicate of [39]	8
71	[71]	Ahuja et al.	2024	Proc. EXPLAINS 2024 Conf.	Cardiac diagnostic datasets	EHR + Imaging + Clinical	Iterative multimodal feature fusion + XAI	Late	NR	NR	NR	Grad-CAM+SHAP	Iterative DL fusion; XAI interpretability; conference paper	7
72	[72]	Thangaraj et al.	2024	European Heart Journal, vol.45	Digital twin clinical datasets	EHR + Imaging + Digital Twin	Digital twin + generative AI	Late	NR	NR	NR	None	Digital twin CVD; generative AI era; European Heart Journal flagship	9

73	[73]	Wani et al.	2024	NR (cited in manuscript)	Lung imaging datasets	Medical Imaging (lung/cardiac)	DeepXplainer (BiLSTM-CNN + XAI)	Intermediate	97.43	0.987	NR	Grad-CAM+LIME	Cross-domain XAI validation; 98.71% sensitivity; lung cancer DeepXplainer	8
74	[74]	Alzahrani et al.	2024	NR (cited in manuscript)	Breast imaging datasets	Medical Imaging (breast/cardiac)	Efficient DL + XAI (BiLSTM-CNN+SHAP)	Intermediate	NR	NR	NR	SHAP	Cross-domain XAI for breast cancer; applicable to cardiac AI	7
75	[75]	Banerjee et al.	2024	NR (cited in manuscript)	65 ML study meta-dataset	Multi-source (meta-analysis)	Pyramidal attention T-network (review)	Review	NR	NR	NR	XAI (general)	65 ML study systematic review; translational gap analysis; brain tumor origin	7
76	[76]	Singh et al.	2024	NR (cited in manuscript)	Diabetic retinopathy datasets	Medical Imaging (retinal)	DL for diabetic retinopathy	Intermediate	NR	NR	NR	None	Cross-domain DL; retinal imaging; applicable to vascular CVD risk	6
77	[77]	Singh et al.	2024	NR (cited in manuscript)	Breast cancer datasets	Medical Imaging (breast)	Enhanced DL for breast cancer	Intermediate	NR	NR	NR	None	Computational approach study; cross-domain XAI	6
78	[78]	Narayan et al.	2024	NR (cited in manuscript)	Prostate imaging datasets	Medical Imaging (prostate)	DL architectures for prostate cancer	Intermediate	NR	NR	NR	None	Cross-domain DL comparison; prostate segmentation	6

3. Extraction Summary Statistics

Extraction Summary Statistics	
**Total studies included**	69 (2012–2025, 1 study 2026)
**Studies with reported accuracy (%)**	~42 / 69 (61%)
**Studies with reported AUC**	~38 / 69 (55%)
**Studies with XAI methods**	~28 / 69 (41%)
**Studies using EHR/Clinical data**	69 / 69 (100%)
**Studies using Medical Imaging**	~31 / 69 (45%)

Extraction Summary Statistics	
**Studies using ECG/Physiological signals**	~29 / 69 (42%)
**Studies using IoT/Wearable data**	~18 / 69 (26%)
**Studies using Genomics/Omics**	~8 / 69 (12%)
**Federated learning studies**	~5 / 69 (7%)
**Top accuracy reported**	99.9% — DEEP-CARDIO (BiGRU-Attention) [8]
**Top AUC reported**	0.999 — DEEP-CARDIO [8]; 0.99 — FL-LSTM+SHAP [67]
**Studies with external validation**	~12 / 69 (17%)
**Studies with prospective trials**	0 / 69 (0%)
**Review/survey articles (no original model)**	~18 / 69 (26%)
**Publication year range**	2012–2026 (59% from 2024–2025)
**Dominant architecture families**	BiGRU-Attention, CNN-LSTM, Federated DL, Transformer
**Dominant fusion strategies**	Late fusion (42%), Attention/Transformer (29%), Intermediate (18%), Early (11%)
**Quality Score range (reviewer)**	5–9 / 10 (mean ~7.0)
**Random seed reporting rate**	~58% of quantitative studies

4. Notes on Data Quality and Limitations

1. Approximately 26% of the 69 entries are review/survey articles that synthesised existing literature rather than presenting original models. These are included as they informed the methodological synthesis but do not contribute performance metrics.

2.. Performance metrics (Accuracy, AUC, F1) were extracted as reported by original authors. Heterogeneous evaluation protocols — different datasets, class ratios, and validation strategies — mean direct cross-study comparison should be interpreted with caution.

4. XAI method documentation was explicitly reported in only ~41% of studies (28/69). For the remaining studies, 'None' indicates the absence of an explainability component, not a data-extraction gap.

5. Quality scores (1c column 'Q') are reviewer-assigned estimates based on: dataset size and diversity (0–2 pts), validation rigor including external validation (0–2 pts), XAI/transparency (0–2 pts), reproducibility/code availability (0–2 pts), and clinical translation evidence (0–2 pts).
